# Comparative genomic analysis of trypanosomatid protists illuminates an extensive change in the nuclear genetic code

**DOI:** 10.1128/mbio.00885-25

**Published:** 2025-04-28

**Authors:** Kristína Záhonová, Zoltán Füssy, Amanda T. S. Albanaz, Anzhelika Butenko, Ambar Kachale, Natalya Kraeva, Arnau Galan, Alexandra Zakharova, Bojana Stojanova, Jan Votýpka, Alexei Y. Kostygov, Viktoria V. Spodareva, Marina N. Malysheva, Alexander O. Frolov, Igor B. Rogozin, Zdeněk Paris, Leoš Shivaya Valášek, Vyacheslav Yurchenko, Julius Lukeš

**Affiliations:** 1 Life Science Research Centre, Faculty of Science, University of Ostrava 48300, Ostrava, Czechia; 2 Institute of Parasitology, Biology Centre, Czech Academy of Sciences 48311, České Budějovice (Budweis), Czechia; 3 Department of Parasitology, Faculty of Science, Charles University, BIOCEV, Vestec, Czechia; 4 Division of Infectious Diseases, Department of Medicine, Faculty of Medicine and Dentistry, University of Alberta 215465, Edmonton, Alberta, Canada; 5 Scripps Institution of Oceanography, University of California San Diego 70015 , La Jolla, California, USA; 6 Faculty of Science, University of South Bohemia 204738 , České Budějovice (Budweis), Czechia; 7 Zoological Institute, Russian Academy of Sciences, St. Petersburg, Russia; 8 Institute of Microbiology, Czech Academy of Sciences 86863, Prague, Czechia; Duke University School of Medicine, Durham, North Carolina, USA

**Keywords:** AT-rich genomes, nuclear genetic code, reassigned codon, tRNA structure, eukaryotic release factors, termination of translation

## Abstract

Trypanosomatids are among the most extensively studied protists due to their parasitic interactions with insects, vertebrates, and plants. Recently, *Blastocrithidia nonstop* was found to depart from the canonical genetic code, with all three stop codons reassigned to encode amino acids (UAR for glutamate and UGA for tryptophan), and UAA having dual meaning also as a termination signal (glutamate and stop). To explore features linked to this phenomenon, we analyzed the genomes of four *Blastocrithidia* and four *Obscuromonas* species, the latter representing a sister group employing the canonical genetic code. We found that all *Blastocrithidia* species encode cognate tRNAs for UAR codons, possess a distinct 4 bp anticodon stem tRNA^Trp^_CCA_ decoding UGA, and utilize UAA as the only stop codon. The distribution of in-frame reassigned codons is consistently non-random, suggesting a translational burden avoided in highly expressed genes. Frame-specific enrichment of UAA codons immediately following the genuine UAA stop codon, not observed in *Obscuromonas*, points to a specific mode of termination. All *Blastocrithidia* species possess specific mutations in eukaryotic release factor 1 and a unique acidic region following the prion-like N-terminus of eukaryotic release factor 3 that may be associated with stop codon readthrough. We infer that the common ancestor of the genus *Blastocrithidia* already exhibited a GC-poor genome with the non-canonical genetic code. Our comparative analysis highlights features associated with this extensive stop codon reassignment. This cascade of mutually dependent adaptations, driven by increasing AU-richness in transcripts and frequent emergence of in-frame stops, underscores the dynamic interplay between genome composition and genetic code plasticity to maintain vital functionality.

**IMPORTANCE:**

The genetic code, assigning amino acids to codons, is almost universal, yet an increasing number of its alterations keep emerging, mostly in organelles and unicellular eukaryotes. One such case is the trypanosomatid genus *Blastocrithidia*, where all three stop codons were reassigned to amino acids, with UAA also serving as a sole termination signal. We conducted a comparative analysis of four *Blastocrithidia* species, all with the same non-canonical genetic code, and their close relatives of the genus *Obscuromonas*, which retain the canonical code. This across-genome comparison allowed the identification of key traits associated with genetic code reassignment in *Blastocrithidia*. This work provides insight into the evolutionary steps, facilitating an extensive departure from the canonical genetic code that occurred independently in several eukaryotic lineages.

## INTRODUCTION

The genetic code, being the molecular dictionary that living organisms use to translate nucleotides into proteins, is a universal feature predominantly represented in its canonical form. However, deviations from the canonical code are observed across various biological systems, including mitochondrial, nuclear, bacterial, plastid, and viral genomes (1–3). Several mutually non-exclusive hypotheses have been posited to explain the causes and mechanisms underlying alterations to the canonical genetic code (4). Mitochondrial genomes in general, and the nuclear genomes of unicellular eukaryotes (protists) in particular, stand out by their capacity to alter the genetic code, with ciliates being especially prominent in this respect (5, 6).

Understanding the *in vivo* malleability and/or plasticity of the genetic code has important consequences for current attempts to generate synthetic genomes with a rewritten and/or expanded code, which would render their bearers bio-containable and resistant to viruses and horizontal gene transfers (7, 8). Furthermore, the incorporation of non-canonical amino acids (aa) via “free” codons will furnish the resulting proteins with novel functions (9). However, the insights gained from naturally evolved non-canonical genetic codes have been scarce, as they are frequently found in organellar genomes with minimal relevance to nuclear genomes, in uncultivable organisms, or in protists with complex and poorly understood genomes that lack closely related species following the canonical code.

Notably, a genetic code with all three stop codons reassigned as sense codons was described in the nucleus of an uncultured kinetoplastid flagellate *Blastocrithidia* sp. ex *Lygus hesperus* (10). Following the establishment of an axenic culture for the related *Blastocrithidia nonstop*, its nuclear genome was sequenced and found to contain over 7,200 predicted protein-coding genes with a non-random distribution of in-frame reassigned codons (11). Unique features of this reassignment include mutations in the eukaryotic release factor 1 (eRF1) massively potentiating readthrough of UGA decoded as tryptophan (Trp) by a special tRNA^Trp^ variant with a shortened anticodon stem (11), which allows specific interactions with protein constituents of the ribosomal A site (12). The UAA and UAG (collectively, UAR) are decoded by newly acquired cognate tRNAs and specify glutamate (Glu). Moreover, in *B. nonstop*, UAA serves as the sole translation terminator, thus having dual meaning (11). Interestingly, although mitochondrial translation in *B. nonstop* depends on tRNAs imported from the cytosol, the non-canonical code of the nuclear genome does not extend to the organelle, resulting in an organism utilizing two distinct non-canonical codes (13).

To dissect these novel features, we have examined the nuclear genomes of four *Blastocrithidia* species and four of their close relatives belonging to the genus *Obscuromonas* (14). Across-genomes comparative analyses of these eukaryotes with either the canonical or the non-canonical nuclear genetic code allowed us to identify novel features associated with, or perhaps even triggering, a wholesale genetic code reassignment.

## RESULTS

### General features of *Blastocrithidia* and *Obscuromonas* spp. nuclear genomes

**Fig 1:**
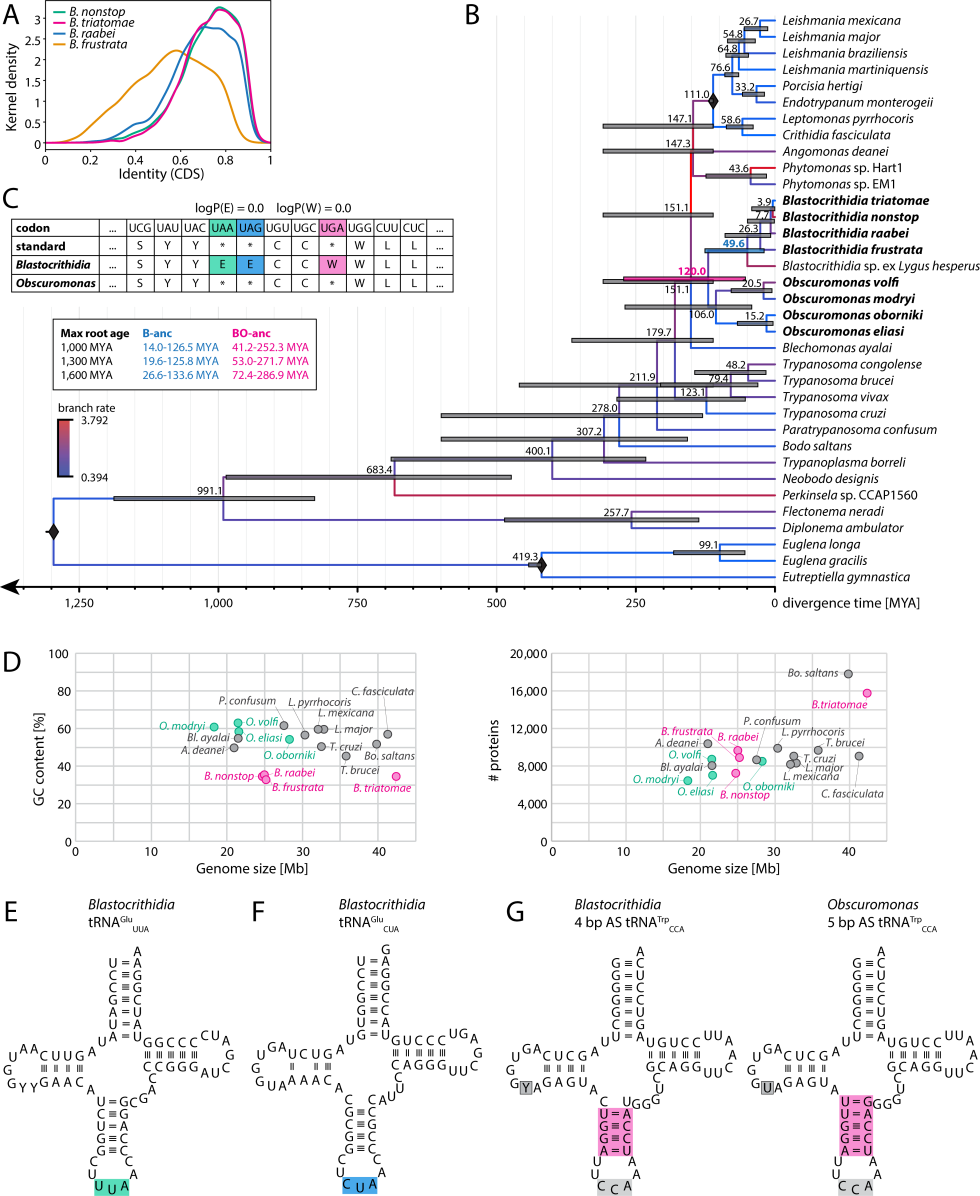
Species, genomes, and genetic codes. (**A**) Kernel density of average identity of coding sequences to their orthologs in other *Blastocrithidia* spp., displaying their relative divergence. (**B**) Phylogenetic timetree of Euglenozoa, with a special focus on Blastocrithidiinae. The underlying data represent a subset of the phylogenomic matrix from the species tree in Fig. S1. The divergence times were determined by RelTime using three calibration points marked as diamonds (Leishmaniinae, Euglenida, and Euglenozoa root). Predicted divergence times are displayed at the nodes, with 95% confidence intervals (CI) as colored bars. In the absence of other calibration points, the Euglenozoa root had a strong effect on internal node ages, and we tabulate the *Blastocrithidia* ancestor (B-anc, blue node label) and *Blastocrithidia*/*Obscuromonas* ancestor (BO-anc, pink node label) CI for the settings used for comparison (1,000 MYA, 1,300 MYA, and 1,600 MYA, that is, minimum CI, mean, and maximum CI for this node as determined previously [23]). The timetree shown was calculated using the 1,300 MYA Euglenozoa root setting. Branch substitution rates are shown as shades of blue-red. Species studied in this work are in bold. (**C**) Part of the genetic code predictions from Codetta (for the full output see Fig. S2). Note that the results for all species of the same genus were identical and are thus simplified here for visualization purposes. ifRCs in *Blastocrithidia* are in colors, and their log decoding probabilities are shown above the table. (**D**) Comparison of the GC content (left) and the number of predicted proteins (right) relative to the genome size in different kinetoplastids. (**E**) tRNA^Glu^_UUA_ recognizing reassigned UAA codons, with the UUA anticodon highlighted in green. (**F**) tRNA^Glu^_CUA_ recognizing reassigned UAG codons, with the CUA anticodon highlighted in blue. (**G**) tRNA^Trp^_CCA_ with a shortened (4-bp-long) AS recognizing reassigned UGA codons in *Blastocrithidia* spp. compared with a canonical 5-bp-long AS in *Obscuromonas* spp. Both tRNAs also recognize UGG codons. Both ASs are highlighted in pink. Differences in the tRNA sequences of *Blastocrithidia* and *Obscuromonas* spp. are shown with a gray background.

Despite the recent substantial increase in the number of sequenced trypanosomatid genomes (15), only one genome of *Blastocrithidia* (that of *B. nonstop*) was scrutinized. The genome of *B. triatomae* has only been investigated for the presence of endogenous viral elements and transposons (16). To enable comparative analyses, we have isolated and introduced into axenic culture *Blastocrithidia raabei* and *Blastocrithidia frustrata* (17, 18). These four species broadly cover the diversity and geographic distribution within this genus (Table 1) and are quite divergent in terms of sequence similarity (Fig. 1A). To allow a wider comparative analysis, we also included members of the closest known lineage represented by the genus *Obscuromonas* (19). The nuclear genome sequence of *Obscuromonas modryi* was reported recently (15), whereas those of *Obscuromonas volfi*, *Obscuromonas eliasi*, and *Obscuromonas oborniki* have been sequenced herein (Table 1). To assess the divergence time between the *Blastocrithidia* and *Obscuromonas* lineages, we built a phylogenetic tree (Fig. S1) using a data set of 240 conserved eukaryotic single-copy genes (20) and including data from the uncultured basal-branching *Blastocrithidia* sp. ex *Lygus hesperus* (10). By applying molecular dating using a local clock model (21), we estimated the divergence of the two genera occurred ~120 million years ago (MYA) (95% confidence interval [CI] 41–287 MYA) (Fig. 1B), whereas the time of radiation of the extant *Blastocrithidia* spp. was ~50 MYA (95% CI 14–134 MYA), placing the emergence of the altered genetic code between these two time points (Fig. 1B).

**TABLE 1:** Information on species used in this study

Organism full name	Information on the isolates used					
	Name	Hemipteran host species (family)	Tissue	Country (locality)	Year of isolation	Isolated by
*Blastocrithidia nonstop* Votýpka et Lukeš, 2023	P57	*Eysarcoris aeneus* (Pentatomidae)	Hindgut	Czechia (Podtrosecké rybníky)	2009	Votýpka
*Blastocrithidia triatomae* Cerisola et al., 1971	Cerisola^*a*^	*Triatoma infestans* (Reduviidae)	Intestinal tract	Argentina	1971	Cerisola et al.
*Blastocrithidia raabei* Lipa, 1966	HR-05	*Coreus marginatus* (Coreidae)	Midgut	Croatia (Žuljana)	2018	Votýpka
*Blastocrithidia frustrata* Malysheva, Ganyukova et Frolov, 2020	4femMK	*Halyomorpha halys* (Pentatomidae)	Midgut + hindgut	Russia (Krasnodarski Krai, Sochi)	2018	Malysheva et al.
*Obscuromonas modryi* Votýpka et Lukeš, 2021	Fi-14	*Riptortus linearis* (Alydidae)	Midgut	The Philippines (Luzon, Bontoc)	2013	Votýpka and Lukeš
*Obscuromonas volfi* Votýpka et Lukeš, 2021	CC-37A	*Catorhintha selector* (Coreidae)	Midgut + hindgut	Caribbean island of Curacao (Souax)	2015	Votýpka and Lukeš
*Obscuromonas eliasi* Votýpka et Lukeš, 2021	PNG-74	*Graptostethus servus* (Lygaeidae)	Malpighian tubules	Papua New Guinea (Nagada)	2011	Votýpka and Lukeš
*Obscuromonas oborniki* Votýpka et Lukeš, 2021	M-09	*Aspilocoryphus unimaculatus* (Lygaeidae)	Midgut	Madagascar (Ambatofosty)	2010	Votýpka and Lukeš

^*a*^ This isolate was obtained from Dr. Günter Schaub in 1996 (originally named only *B. triatomae*; see reference 22). Previously published sequences AF153037 and KX138599 are from the same strain.

Three short read assembly methods were compared to produce robust assemblies (Materials and Methods; Table S1A and B). *Blastocrithidia* spp. genome assembly sizes were ~25 Mb, except for *B. triatomae,* which has a much bigger size (42 Mb), representing the only outlier in our data set. The sizes of *Obscuromonas* spp. genome assemblies also varied, with those of *O. volfi* and *O. eliasi* ~21 Mb in length, and those of *O. modryi* and *O. oborniki* 18 Mb and 28 Mb, respectively. The nuclear genomes of all *Blastocrithidia* spp. proved to be GC-poor (33%–36%; extremely low values for kinetoplastid parasites [Fig. 1D]), whereas those of *Obscuromonas* spp. (54%–63%; Table S1B) resembled other trypanosomatid genera (15). Importantly, in all *Blastocrithidia* spp., UAR and UGA specify Glu and Trp, respectively, whereas no such deviation was observed for *Obscuromonas* spp. (Fig. 1C; Fig. S2), documenting the use of the canonical genetic code in this sister lineage.

Assembly completeness was assessed by BUSCO using the genome-derived proteomes (Fig. S3A; Table S1C), with in-frame reassigned codons in *Blastocrithidia* spp. translated as their corresponding aa. The percentage of missing genes spanned 5%–12% and 40%–45% when the Euglenozoa and broader Eukaryota data sets were used, respectively. Notably, a high proportion of the *B. triatomae* and *O. oborniki* proteins was represented by duplicated markers (46.2%/31.8% and 16.9%/11.4% in the Euglenozoa/Eukaryota data sets, respectively), whereas other species possessed fewer duplicated BUSCOs (<3%/≤2% in the Euglenozoa/Eukaryota data sets). In *B. triatomae*, the elevated rate of duplications was accompanied by a high number of total predicted proteins (15,768). This is usually indicative of contamination, but analysis of 18S rRNA genes confirmed the exclusive presence of *B. triatomae* or *O. oborniki* in the respective genomic data. In *B. triatomae*, duplicated genes and contigs exhibited similar raw read coverage as their single-copy counterparts (Fig. S3B), which is compatible with partial genome duplication, a phenomenon also documented in other trypanosomatids (24). In *O. oborniki*, however, a similar gene coverage analysis suggests duplications could be, to some extent, allelic variants or assembly artifacts (Fig. S3B). Altogether, the genome and proteome sizes in both genera are similar to those of other kinetoplastids (Fig. 1D).

Since approaches with and without prior ploidy assumption provide the same results (15), we next estimated the somy levels for 100 longest scaffolds (used as chromosome proxies) assuming that the median genome coverage reflects a disomic state (Table S2). We extended the analysis to all Blastocrithidiinae (i.e., *Blastocrithidia* and *Obscuromonas*) species and evaluated the prevalence of the disomic state with varying degrees of aneuploidy, previously demonstrated for several trypanosomatids (15, 25). Only *B. triatomae* and *O. volfi* lack any monosomic scaffolds, whereas in general, monosomy and trisomy were the most common states after disomy, with only a few scaffolds surpassing trisomy (Table S2).

### The machinery for decoding reassigned codons

The number of predicted tRNA genes per genome varies (Table S3), with the lowest (70) and highest (95) numbers documented in *B. nonstop* and *B. triatomae*, respectively, which are in the range (58–120) documented in trypanosomatids (26). The tRNAs cognate to UAR were prominently present in all *Blastocrithidia* spp. and absent from all *Obscuromonas* spp. (Fig. 1E and F; Table S3). The only exception was *B. raabei*, where only the 3'-terminal part of the gene for tRNA^Glu^_UUA_, which decodes UAA, was retrieved. However, this corresponds to an assembly gap, since the full-length sequence was reconstructed from reads, and the identity of this tRNA was confirmed by Northern blotting (Fig. S4). The tRNA^Trp^_CCA_, with an anticodon stem (AS) exhibiting a structure critical for UGA decoding—specifically, a 4 bp stem caused by a mismatch loosening the top pair of the canonical 5-bp-long AS (11)—was exclusively identified in *Blastocrithidia* spp., whereas *Obscuromonas* spp. encoded the canonical AS variant (Fig. 1G).

**Fig 2:**
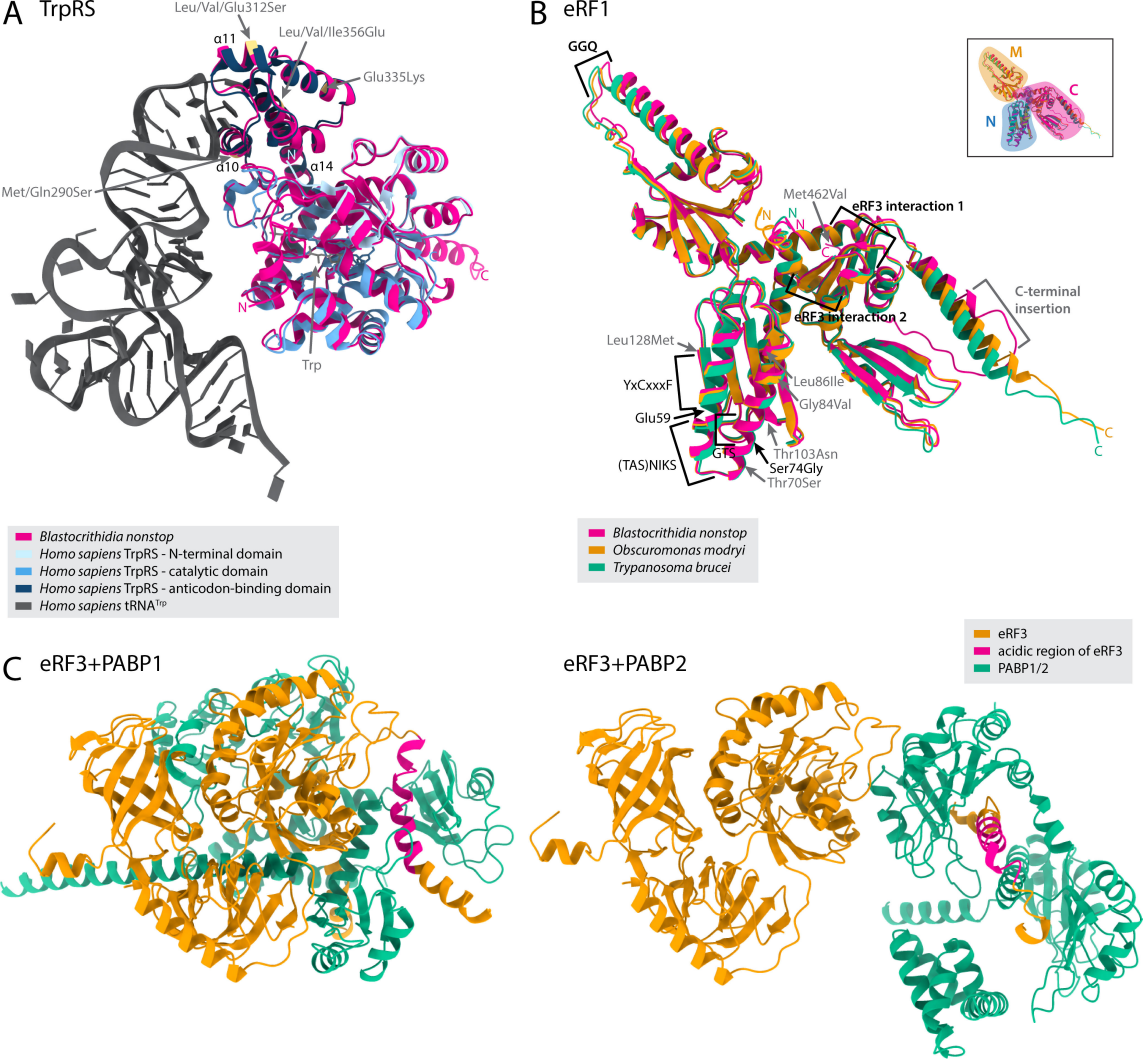
Predicted structures of selected proteins. (**A**) Structural alignment of *B. nonstop* and *H. sapiens* TrpRS models, with N- and C-termini of the respective proteins marked in the same colors. The *B. nonstop* model was predicted by AlphaFold; the *H. sapiens* structure in complex with tRNA^Trp^ was determined experimentally (28). The four mutations from Fig. S5 are shown in yellow in the *B. nonstop* structure, the three α-helices forming a pocket accommodating the anticodon loop are marked in black. (**B**) Structural alignment of *T. brucei*, *B. nonstop*, and *O. modryi* eRF1 models predicted by AlphaFold, with N- and C-termini of the respective proteins marked in the same colors. Functional motifs from Fig. S10 are annotated in black, and the UGA readthrough-increasing mutation Ser74Gly is found in the proximity of the (TAS)NIKS motif of the N domain. Other mutations in less conserved regions are in gray. The inset shows the three major domains of eRF1 (29). (**C**) Structure of *B. nonstop* eRF3 in complex with PABP protein (PABP1 left, PABP2 right) as predicted by AlphaFold. Some unstructured loops were omitted for visualization purposes.

Although tRNA^Trp^_CCA_ has an altered structure in *Blastocrithidia* spp., it must be recognized by tryptophanyl-tRNA synthetase (TrpRS) to be charged with Trp. In trypanosomatids, two such enzymes exist, namely, TrpRS1 charging the cytosolic tRNA^Trp^ and TrpRS2 charging tRNA^Trp^ upon its import into the mitochondrion (27), with *Blastocrithidia* spp. retaining both (Table S4). We noticed four substitutions in the anticodon-binding domain of TrpRS1 proteins of all *Blastocrithidia* spp., at positions generally conserved in kinetoplastids (Fig. S5). When the predicted *B. nonstop* TrpRS1 structure was overlaid onto the experimentally determined structure of human TrpRS in complex with tRNA^Trp^ (28), two of the uniquely substituted residues, Met/Gln290Ser and Leu/Val/Ile356Glu (positions in the *B. nonstop* sequence), were found in the proximity of the “anticodon arm recognition” and “anticodon recognition” motifs, respectively (Fig. 2A). The other two substituted positions were in more distant helices of the C-terminal domain. Leu/Val/Glu312Ser was part of helix α11 (307–313), and Glu335Lys was opposite to helices α10 (288–298), α11, and α14 (350–374) that collectively form a pocket accommodating the anticodon loop.

In eukaryotes, in-frame UGAs can be recognized by a specific selenocysteine (Sec) tRNA, when guided by the Sec insertion sequence (SECIS) element that enables the incorporation of Sec into a polypeptide (30). Five proteins involved in Sec synthesis and incorporation, and three selenoproteins (SelK, SelT, and SelTryp) were previously identified in kinetoplastids (31, 32). Notably, tRNA^Sec^_UCA_ and other components of the Sec utilization toolkit are present in all Blastocrithidiinae species (Fig. S6; Table S5). Homologs of SelK, SelT, and SelTryp with the in-frame UGAs were found in all *Blastocrithidia* spp., whereas SelK possessed in-frame UGA only in *O. eliasi*, and SelTryp was missing in *O. modryi* and *O. volfi*. The high degree of similarity of these homologs with verified selenoproteins of *Trypanosoma brucei* and *Leishmania major* makes the presence of Sec insertion likely in Blastocrithidiinae (Fig. S7). No selenoproteins known from other organisms were identified (Table S5). This documents that the UGA codon is used to encode two different aa in a position-specific manner (Fig. S8).

### Usage of in-frame reassigned codons

**Fig 3:**
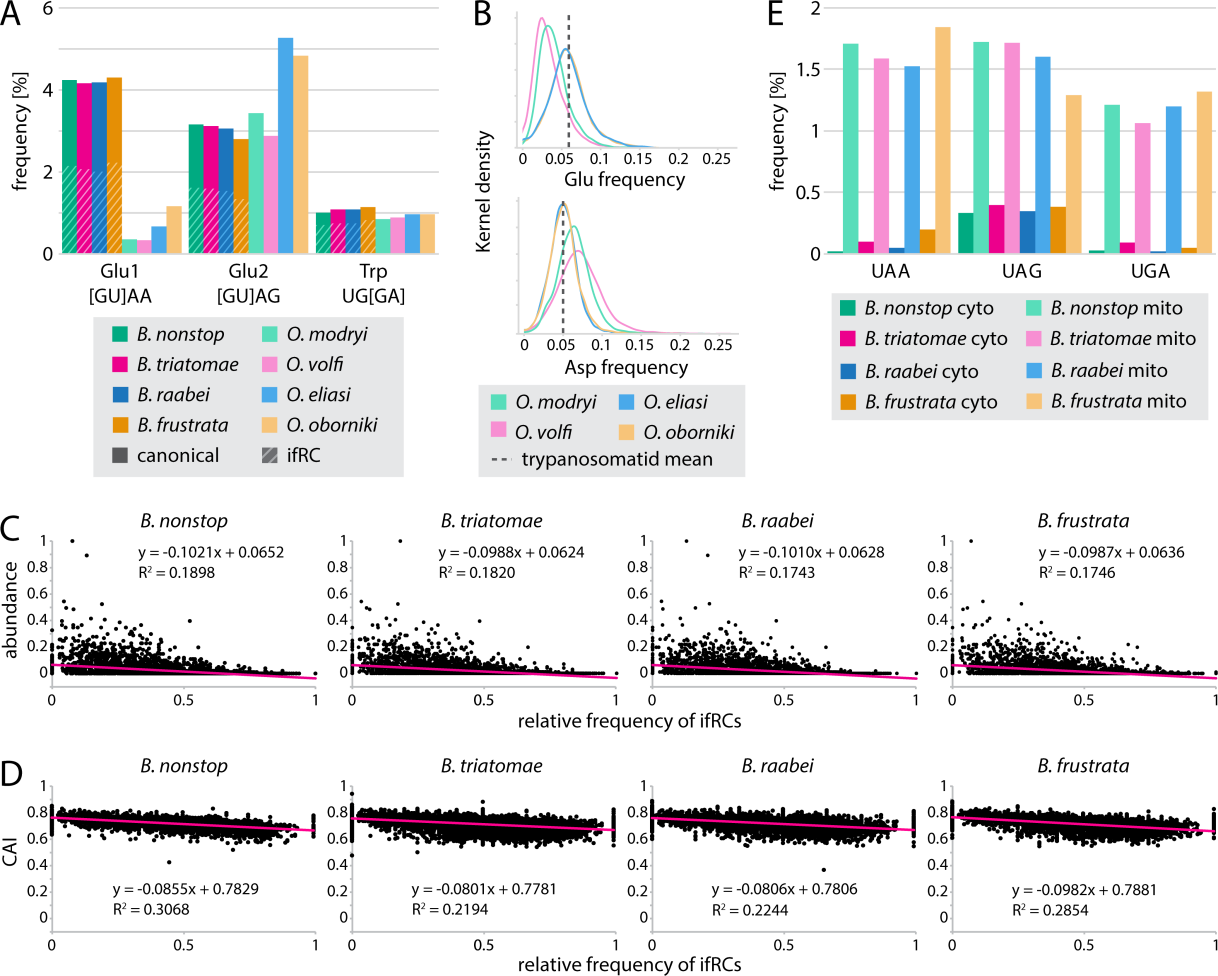
Codon usage in Blastocrithidiinae. (**A**) Usage of Glu and Trp codons in Blastocrithidiinae. Number of used codons was normalized to total codon count of all CDSs: Glu1 = (GAA + UAA)/codons; Glu2 = (GAG + UAG)/codons; Trp = (UGG + UGA)/codons. The proportion of ifRCs is shaded for *Blastocrithidia* spp., whereas no such codons were recorded for *Obscuromonas* spp. (**B**) The Glu depletion in *O. modryi* and *O. volfi,* as shown in panel A, is mirrored by the enrichment of Asp, an acidic side chain amino acid with similar physicochemical characteristics as Glu. Glu and Asp codon frequencies in all CDSs are shown as distribution density plots; the distribution of the sum of Glu and Asp codons is similar for all Blastocrithidiinae species (not shown). The mean Glu and Asp content in selected trypanosomatids (data from VEuPathDB), shown by dashed lines, suggests that *O. modryi* and *O. volfi* are uniquely among them Glu depleted and Asp enriched. (**C**) Scatter plots showing the non-random distribution of ifRCs in proteins (black dots) based on protein MS data. The *x*-axis shows the relative frequency of ifRCs, i.e., (UAA + UAG + UGA)/(GAA + UAA + GAG + UAG + UGG + UGA). The *y*-axis unit shows the MS-based relative abundance of *B. nonstop* proteins (in the other three species assumed to be the same for orthologous proteins). The trend line (pink) was generated by linear regression. (**D**) Scatter plots showing the non-random distribution of ifRCs in proteins (black dots) based on codon adaptation index (CAI). The *x*-axis shows the relative frequency of ifRC as above. The *y*-axis unit shows the calculated CAI of *B. nonstop* proteins. The trend line (pink) was generated by linear regression. (**E**) Bar plots showing the high frequency of ifRC in genes for mitochondrial (mito) ribosomal proteins and their relative depletion in highly expressed genes for cytosolic (cyto) ribosomal proteins. The number of ifRCs was normalized to the total codon count of either cytosolic or mitochondrial ribosomal protein genes.

Knowing that all *Blastocrithidia* species encode tRNAs recognizing in-frame reassigned codons (ifRCs), we investigated their usage and calculated a fraction of each codon in their coding sequences (CDSs) (Table S6). The frequency of UAA was the highest (2.06%–2.28%) from all three ifRCs, followed by UAG (1.38%–1.66%), whereas UGA was the least employed codon (0.72%–0.85%) (Fig. 3A). The usage of UGA mirrors that of the aa it encodes, with Trp codon frequencies (UGG + UGA for *Blastocrithidia* and UGG for *Obscuromonas* spp.) comparable in all species (Fig. 3A). Next, we divided Glu codons into two categories according to their third position (11): Glu1 stands for GAA and UAA, and Glu2 for GAG and UAG codons. Note that no in-frame UAA and UAG were found in *Obscuromonas* spp. The usage of the Glu1 codons was comparable among *Blastocrithidia* spp. (4.28%–4.41%) and much lower in *Obscuromonas* spp. (0.35%–1.19%), which correlates with the differences in the GC content between the genera. The Glu2 codons were used at similar frequencies not only in *Blastocrithidia* spp. (2.88%–3.25%) but also in *O. modryi* (3.52%) and *O. volfi* (2.96%), whereas their usage was much higher in *O. oborniki* (4.96%) and *O. eliasi* (5.42%) (Fig. 3A). In the former two species, the lower frequency of Glu codons mirrors the higher frequency of codons for the other negatively charged aa, aspartate (Asp) (Fig. 3B). Relative to other trypanosomatids, *O. modryi* and *O. volfi* proteins appear Glu-depleted and Asp-enriched (Fig. 3B).

We next investigated whether the ifRC frequency exhibits any biases. We used the previously reported *B. nonstop* mass-spectrometry (MS) data (11), assigned abundance values of *B. nonstop* proteins to their putative orthologs (reciprocal best BLAST hits) in other *Blastocrithidia* species, and correlated these values with the ifRC frequency, that is, (UAA + UAG + UGA)/(GAA + UAA + GAG + UAG + UGG + UGA) (Table S7). We also inferred optimized codon frequencies from the highly abundant *B. nonstop* proteins and calculated the codon adaptation indices of all CDSs as their level of deviation from the optimal codon usage. We consistently detected a negative correlation between ifRC frequency and both the predicted protein abundance and the codon adaptation index (Fig. 3C and D). Next, we calculated the ifRC frequency in sets of nucleus-encoded cytosolic and mitochondrial ribosomal proteins. Few ifRCs were found in the genes for cytosolic ribosomal proteins (0.02%–0.39%), whereas their frequencies in their mitochondrial counterparts were significantly higher (1.06%–1.84%) (Fig. 3E). To address whether there is a pronounced codon usage bias when these two gene categories are compared, we made the mitochondrial vs. cytosolic ribosomal proteins comparison for all codons, including sequences from *Obscuromonas* spp. and *T. brucei* as control. A few other codons showed similar but much weaker trends (AGA in *Blastocrithidia* spp. and UGU, UUU, and AUA in all species analyzed) (Fig. S9). Hence, the usage of ifRCs seems under control by selection, presumably because these codons have a strong impact (among other codons) on translation efficiency and/or accuracy.

We also investigated whether various functional protein categories have different ifRC frequencies, that is, UAA% = UAA/(UAA + GAA) × 100 for Glu1, UAG% = UAG/(UAG + GAG) × 100 for Glu2, and UGA% = UGA/(UGA + UGG) × 100 for Trp. Overall, Trp codons were most frequently represented by the corresponding ifRC, and this frequency was strongly function-dependent, as supported by pairwise comparisons (Fig. S10). Functional protein groups annotated as energy production and conversion (C), aa transport and metabolism (E), nucleotide transport and metabolism (F), translation, ribosomal structure and biogenesis (J), and cell motility (N) had the lowest ifRC frequencies, whereas functional groups of cellular processes and signaling, cell cycle control, cell division, chromosome partitioning (D), and those comprising proteins with unknown function (S) and unannotated genes (-) showed the highest ifRC frequencies (Fig. S10A). The UAR Glu codons showed less frequent ifRCs compared with the UGA Trp codon, although differences among functional groups remained significant (Fig. S10B). Differences among the Glu2 codon frequencies were least pronounced, suggesting these substitutions have a more neutral effect compared to Glu1.

**Fig 4:**
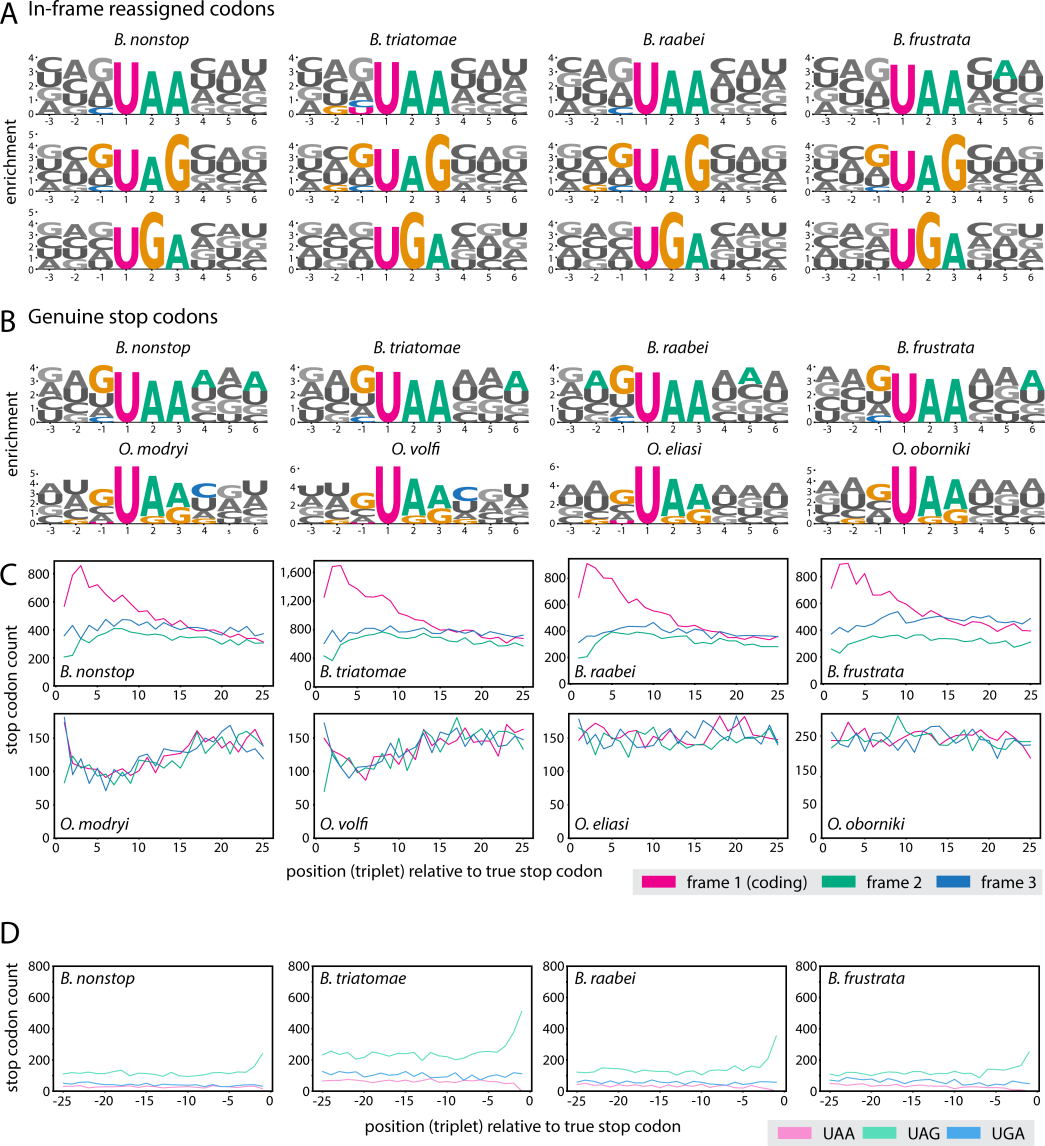
Genomic context of stop codons. (**A and B**) Background-normalized logos of ifRCs in *Blastocrithidia* spp. (**A**) and genuine stop codon flanking sequences in Blastocrithidiinae (**B**). The enrichments were calculated as normalized to the average nucleotide content of all coding sequences, which is why A appears relatively enriched compared with G in *Obscuromonas* spp. stop codons, although UAG and UGA are more prevalent (compare with Fig. S13). Significant enrichments are shown in color, non-significant in shades of gray. (**C**) Summary counts of true stop codons (UAA for *Blastocrithidia* spp., all three canonical stops for *Obscuromonas* spp.) in 25 triplets after the genuine (translation-terminating) stop codon, in three coding frames, 1 being the protein-coding frame. (**D**) Counts of all stop codons in 25 triplets before the genuine stop codon in *Blastocrithidia* spp.

To understand the genomic context of *Blastocrithidia* ifRCs, we analyzed nucleotides flanking each ifRCs (Fig. 4A). In the case of UAR codons, we often found an enrichment of G (in UAG codons) and depletion of C before (in UAR codons) at position −1 of these codons. However, no change was observed for UGA.

### Release factors and termination of translation

A single protein, eRF1, is responsible for recognizing all three stop codons during translation in the canonical genetic code. Several motifs in eRF1 are known to be important for stop codon recognition, that is, GTS, Glu55, (TAS)NIKS, Ser70, and YxCxxxF (residues with human sequence numbering) (33), and mutations in some of these motifs were associated with a narrowed codon specificity of eRF1 in eukaryotes with stop codon reassignments (34–36). In *Blastocrithidia* spp., the UGA readthrough was associated with the Ser70Gly substitution (position 74 in *Blastocrithidia* sequences) (10), and, in combination with the 4-bp-long AS tRNA^Trp^_CCA_, shown to massively potentiate UGA readthrough in a heterologous system (11). Reassuringly, this critical Ser74Gly substitution is invariably present in all *Blastocrithidia* spp. and absent in all other kinetoplastids (Fig. S11). Sequence alignment also revealed six additional positions that are fully conserved in kinetoplastids but substituted in *Blastocrithidia* spp. Although all functional motifs, including both eRF3 binding signatures (29), are conserved in *Blastocrithidia* spp. (Fig. 2B), five of six substitutions confined to members of this genus map to two antiparallel helices harboring all stop codon recognition motifs (Fig. 2B). Moreover, there is an insertion of four to seven aa in the very C-terminal helix in eRF1 of *Blastocrithidia* spp. These changes may be part of a mechanism ensuring that only UAA at the very end of CDSs is recognized as a termination codon in *Blastocrithidia* spp. (see below).

There are almost 30 positions associated with stop codon recognition in model yeasts (37), and one of them, Gly357, is uniquely changed to Ser in *Blastocrithidia* spp. Moreover, two additional residues in *Blastocrithidia* spp. were altered in positions conserved in kinetoplastids and yeast (Fig. S12A). Importantly, the Gln- and Asn-rich N-terminal domain of the yeast eRF3, known for its prion-forming ability that enhances translational readthrough of stop codons (38), is also present in all kinetoplastids (Fig. S12B). However, in all *Blastocrithidia* spp. it is significantly extended and immediately followed by an extremely acidic aa-rich region (comprised primarily of Asp and Glu; Fig. S11A), which is predicted to form close contact with both identified polyA-binding proteins (PABPs) (Fig. 2C; Table S4).

### Genuine stop codons

To identify genuine stop codons, we performed BLAST searches using *B. nonstop* proteins as queries against the genome assemblies of other *Blastocrithidia* spp. and analyzed the identity of the following codon after the end of the alignment with complete 3' ends (see Materials and Methods). The only stop codon terminating translation in all examined *Blastocrithidia* spp. is UAA (Table S8). To investigate whether a bias toward UAA is manifested in the sister genus *Obscuromonas*, we calculated stop codon usage in CDSs of predicted proteins with a complete 3′-end and found that in all *Obscuromonas* spp., the most frequently used termination codon is UAG (Fig. S13). Notably, UAA is the least frequently used termination codon in all species except *O. oborniki*, where UGA is employed least frequently. This aligns with the genome of this species being the most AU-rich within the *Obscuromonas* lineage (Fig. S13; Table S8).

**Fig 5:**
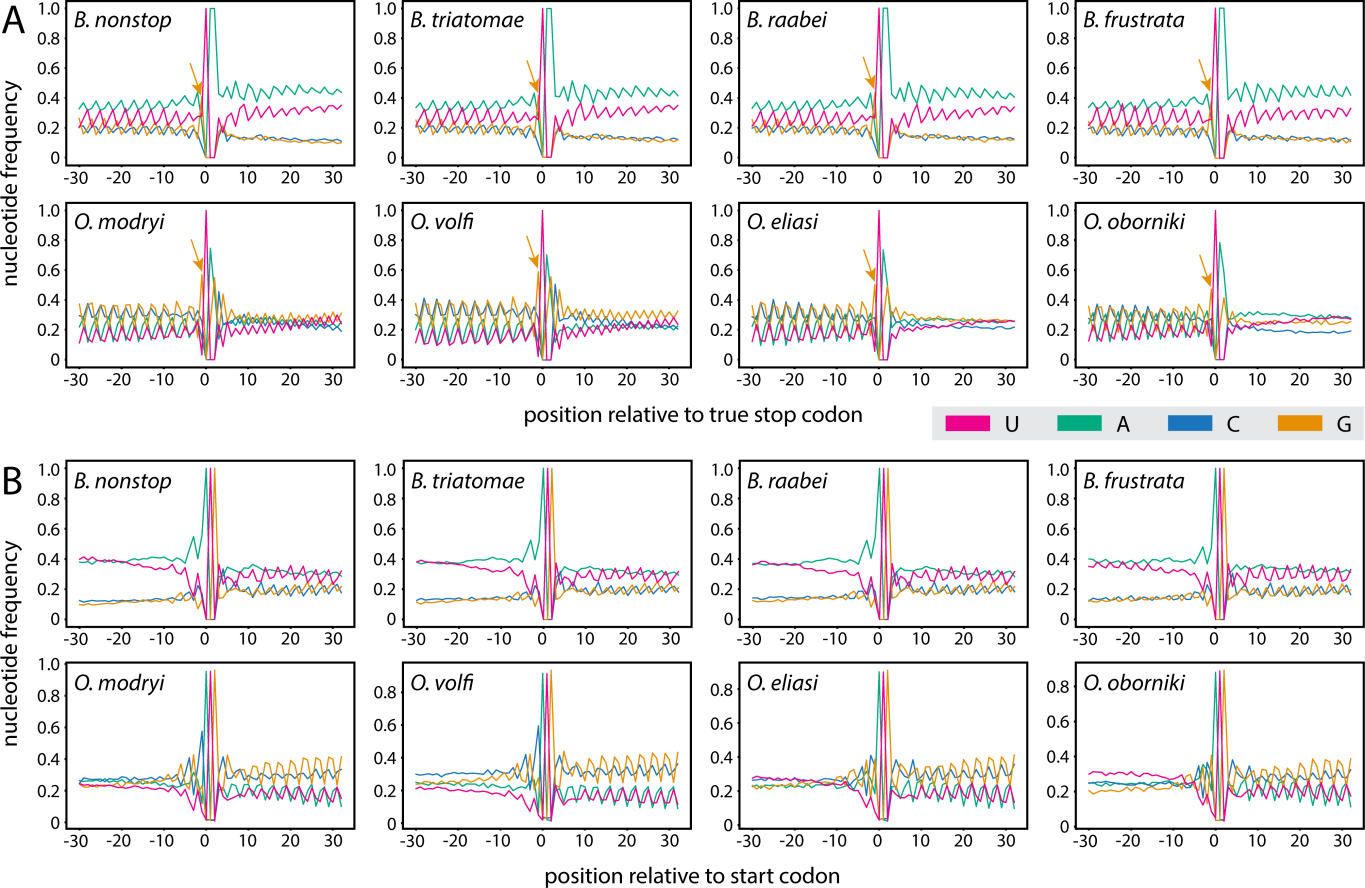
Nucleotide frequency around stop and start codons. Nucleotide frequency up- and down-stream from the genuine stop (**A**) and start (**B**) codon (position 0). The arrows mark the overrepresentation of G right before the genuine stop codon. Although the region upstream of the start is AU-rich, note a balanced frequency of A and U, with little to no zig-zag patterning typical for regions downstream from genuine stops.

Next, we investigated the occurrence of additional stop codons downstream of the genuine stop codon (Fig. 4C). In *Blastocrithidia* spp., UAA was overrepresented until the 13th–15th codon past the genuine stop and, importantly, only in the reading frame of the encoded protein. This overrepresentation is function-independent (Fig. S13). No such trend was seen for *Obscuromonas* spp., where the occurrence of all three stop codons was comparable in all three coding frames and, thus, apparently random. In addition to tandem UAA stop codons, there is an enrichment in A at various positions following the UAA stop codons limited to *Blastocrithidia* spp. (Fig. 4B). The occurrence of ifRCs −25 codons upstream of the genuine stop codon appears to be on background level, with the exception of UAG in positions −2 and −1, which likely represents proteins encoding Glu at their very C terminus (Fig. 4D). Additionally, although *Blastocrithidia* spp. uniquely exhibit AU-rich UTRs, their 3' UTRs display a distinct zig-zag pattern of A and U distribution, contrasting with the more balanced AU frequencies observed in the 5' UTRs (Fig. 5A and B). This pattern does not seem to stem from codon bias, as it is restricted to A and U nucleotides and is primarily observed in sequences that contain additional UAA codons within their presumed 3' UTRs. We also noticed an enrichment of G directly preceding the stop (−1 position) in both Blastocrithidiinae lineages (Fig. 5A) that might further prevent readthrough (39).

### Influence of GC content on ifRC usage and their changes in the evolution of *Blastocrithidia*

To understand how the presence of ifRCs is determined by the GC content in coding regions, we estimated the following values: GC content within ORFs, GC content at 4-fold degenerate sites (4fds), and proportions of ifRCs among the codons for Trp and Glu, for sites where these amino acids were conserved across *Blastocrithidia* and *Obscuromonas* spp. (Fig. S15A; Table S9). The distribution of the assessed values in the phylogenetic tree demonstrated a decrease of the GC content in ORFs during the evolution of the genus *Blastocrithidia*, with *Blastocrithidia* sp. ex *Lygus hesperus* displaying the highest value (46.4%) (Fig. S15A). The smallest GC content in ORFs (38.7%) was observed in *B. frustrata* (Fig. S15A). Unexpectedly, *Blastocrithidia* sp. ex *Lygus hesperus* showed the second smallest value for the GC content in 4fds (43.0%). Accordingly, no correlation was observed for the GC content in ORFs and 4fds for *Blastocrithidia* (Fig. S15B, top left panel). However, when considering both *Blastocrithidia* and *Obscuromonas* spp., for which both values were approximately 1.5–2.0 larger, a strong and statistically significant correlation was detected (Fig. S15B, top right panel), which is in line with our a priori assumptions. Of note, the dispersion of values for *Obscuromonas* is rather small. Such a discrepancy suggests that the overall GC content in ORFs in *Blastocrithidia* is no longer the main factor determining the GC content in 4fds.

The proportions of ifRCs showed extreme values in *Blastocrithidia* sp. ex *Lygus hesperus*. It contains only 19.2% of Glu codons, displaying a large difference (13.1%) when compared with the next smallest one, whereas for Trp codons, the percentage was the largest (48.5%) and differed from the neighboring value only by 3.5%. Notably, the corresponding opposite extremes were observed for one of the crown species, *B. triatomae*. These observations indicate that the dynamics of substitutions in the Trp and Glu sites were uncoordinated and non-uniform in the evolution of the *Blastocrithidia* lineage. The two types of ifRCs also showed an essential difference with respect to their relationship with GC content in the coding regions. Proportion of the UAR codons demonstrated an almost absolute and highly statistically significant negative correlation with GC content (Fig. S15B, bottom left panel), whereas in the case of UGA codons, the correlation was weaker, positive, and statistically insignificant (Fig. S15B, bottom right panel).

### Gene family evolution of *Blastocrithidia*

To identify unique genes and putative new functionalities, we investigated the gene content differences between *Blastocrithidia* spp. and other kinetoplastids. A total of 196,129 annotated proteins of 21 kinetoplastids were clustered into 10,219 orthologous groups (OGs) with 227 OGs containing only one species and only ~9% of genes remaining unassigned singletons (Table S10). We then conducted a genome-wide analysis of gene gains and losses, as well as gene family expansions and contractions, along the trypanosomatid phylogeny (Fig. S16). Similarly to internal nodes of the trypanosomatid phylogeny in general, the *Blastocrithidia* common ancestor node showed ~2× more gene losses over gains. Conversely, gene family expansions in the *Blastocrithidia* stem lineage dominated over contractions 8×, thus exhibiting the highest ratio among all nodes examined (Table S11). Of 175 expanded OGs (Table S12), several were involved in DNA replication and repair, ribosome biogenesis, and membrane trafficking, but most were of unknown function. To identify genes associated with the genetic code reassignment, we examined 200 OGs gained and 700 OGs lost at the *Blastocrithidia* node, yet a vast majority of them was annotated as hypothetical (Table S12).

## DISCUSSION

Our comparative analysis reveals that the examined *Blastocrithidia* species share unique genomic features associated with the wholesale stop codons reassignment, which likely occurred in their common ancestor through a cascade of interdependent steps, rendering the extensively altered genetic code stable. The absence of even subtle differences or intermediate stages supports the assumption that this reassignment is old and very stable. This combination of features distinguishes the non-canonical code from other molecular oddities for which trypanosomatids are widely known, such as RNA editing and complex mitochondrial DNA, all subject to tinkering and species-specific alterations (40–42). The singularity of the non-canonical genetic code in *Blastocrithidia* also contrasts with the recurrent evolution of different code variants, including those with all three stop codon reassigned, in multiple lineages of ciliates, pointing to a common “preadaptation” in their ancestor (6, 43–45).

The mechanism of codon reassignment has been explained by different hypotheses. The “codon capture” theory, assuming that the codon first disappears from CDSs to reappear with a new meaning that is captured by a near-cognate tRNA (46), was described in *Escherichia coli* (47). The “ambiguous intermediate” theory proposes that the mutations in tRNA weakening its specificity may accelerate reassignment of a near-cognate codon (48). Although thought to operate in yeast *Candida* (49), this theory was replaced by the “tRNA-loss driven codon reassignment” hypothesis (50) that postulates that the loss of a cognate tRNA allows capturing of the corresponding codon by a near-cognate tRNA (4). Finally, the possibility that the genetic code can be altered by selection shall also be considered. Indeed, the recently described parallel loss of tRNA^Leu^_CAG_ in several yeast lineages in response to a plasmid-encoded killer toxin similar to zymocin that specifically cleaves this tRNA species implies such a scenario (51).

**Fig 6:**
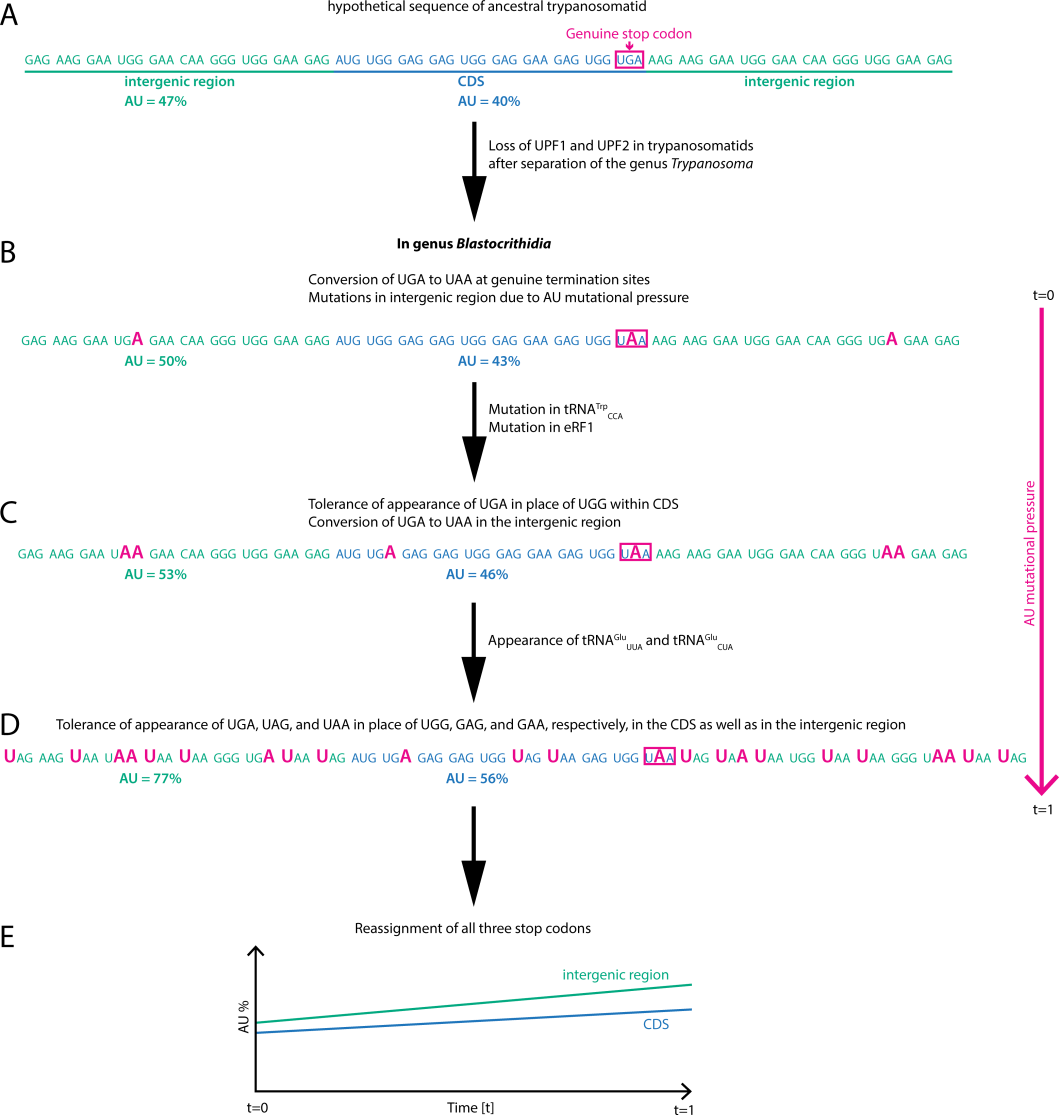
Evolutionary path of genome reassignment as observed in extant *Blastocrithidia* spp. (**A**) The hypothetical gene unit of an ancestral trypanosomatid consists of a coding sequence (CDS) shown in blue, surrounded by an intergenic region depicted in green. (**B**) Early in trypanosomatid evolution, the NMD pathway was lost. As a result of AU-biased mutational pressure that (for undefined reasons) started to affect the lineage leading to *Blastocrithidia* (after the divergence of *Obscuromonas*), the GC-rich stop codons UGA and UAG were substituted with UAA, and thus lost the terminating function. (**C**) The continuation of AU-biased mutational pressure substituted the canonical tryptophan codon UGG with UGA. Concurrently, two molecules acquired mutations: in eRF1, they resulted in a decreased affinity toward UGA, whereas in tRNA^Trp^_CCA_, they enabled UGA recognition and capture within the CDS. (**D**) Simultaneously or subsequently, because of the same mutational pressure, the common ancestor of *Blastocrithidia* spp. acquired mutations in one of the duplicated copies of tRNA^Glu^_UUC_ and tRNA^Glu^_CAC_, leading to the emergence of tRNA^Glu^_UUA_ and tRNA^Glu^_CUA_. The ongoing AU mutational pressure turned the canonical glutamate GAA and GAG codons into UAA and UAG, respectively. These could ever since be decoded by the newly emerged tRNA^Glu^, leading to the complete reassignment of all three stop codons.

One or a combination of these mechanisms may explain the genetic code reassignment also in *Blastocrithidia* species. In this lineage, the mechanisms underlying the reassignment of all stop codons—such as the emergence of “suppressor” tRNAs, specific alterations of tRNA^Trp^_CCA_, and mutations in eRF1 and eRF3—are identical, despite the apparent divergence of orthologous genes in these organisms. Following the most parsimonious scenario, this genetic code emerged due to a strong and/or persistent directional mutational pressure (52) in the *Blastocrithidia* stem lineage causing a whole-genome GC content decrease and hence the depletion of UAG and UGA stop codons, in turn allowing additional changes to the termination of translation. The same mutational pressure promoted the conversion of the standard Glu (GUR) and Trp (UGG) codons to UAR and UGA. At some point, the Ser67Gly mutation in eRF1 and shortening of the anticodon stem of tRNA^Trp^_CCA_ to 4 bp hindered efficient recognition of UGA, which could be reassigned to encode Trp, and the evolution of tRNA^Glu^ facilitated the recognition of UAR codons (Fig. 6). The genomic context is enriched for G in the UAG codons and depleted for C in both UAR codons in −1 position (Fig. 4A). However, our across-the-genomes analysis also showed that the UGA codon readthrough is the same for all four UGA-N tetranucleotides (Fig. 4A), further implying the existence of different mechanisms of recognition of this ifRC. Ultimately, AT-rich genomes may offer evolutionary advantages by reducing energetic and nitrogen costs and enabling faster evolution due to increased mutability (see Supplementary Discussion in reference 11 for details). These traits can be particularly beneficial for unicellular eukaryotes adapting to resource-limited environments or evading host defenses.

A peculiar feature of the UGA codon is that in organisms containing selenoproteins, it may be homonymous, that is, specify alternatively a stop codon and the 21st amino acid selenocysteine (53). Furthermore, it was shown in ciliates and a dinoflagellate that the dual role of UGA may extend into the incorporation of two amino acids, namely, Cys and Sec or Trp and Sec (44, 54, 55). Trypanosomatids are also known to contain at least three selenoproteins (31, 32), and *Blastocrithidia* spp. are no exception. Notably, we have found that in one of their selenoproteins, SelTryp, UGA specifies both Trp and Sec, thus being homonymous for these two amino acids.

Comparative analyses revealed AU-rich regions downstream of the genuine stop codons, a trait unique to the genus *Blastocrithidia*, which may facilitate interaction with dedicated RNA-binding proteins to fine-tune translation termination. We speculate that the evolutionarily conserved ability of the PABPs to bind both poly(A) tails and AU-rich RNA molecules (56) has shifted toward binding the AU-rich tails of mRNAs in *Blastocrithidia* spp. This would promote the interaction of PABP with the termination complex, particularly with eRF3, as was demonstrated in opisthokonts (57), to assist in termination at the genuine stop codon. Alternatively, PABP might interact with the negatively charged C-terminal helix of eRF1 that mimics an RNA molecule. In this context, it is important to reiterate that, similarly to karyorelictean ciliates and the heterotrichean ciliate *Condylostoma magnum* with all stop codons reassigned and used as genuine stop codons at the same time (44, 58), the UAA codon in *Blastocrithidia* species is rare before the genuine stop codons, whereas its frequency increases downstream of it (Fig. 4D). All these features may both ensure the recruitment of RNA-binding and termination factors and minimize undesirable readthrough by the newly evolved tRNA^Glu^_UUA_, which is fully cognate to UAA and, thus, represents a strong competitor for eRF1. At the same time, the occurrence of these motifs only downstream of the genuine UAA stop codon would mitigate premature termination on the in-frame UAA codons. A combination of these features, emerging from our comparative analyses, seems to form a robust framework that ensures the translation of the most highly expressed proteins.

Compared with *Obscuromonas* spp., their close relatives now shown to utilize the canonical genetic code, members of the genus *Blastocrithidia* have evolved several modifications in their eRF1, including the critical Ser74Gly substitution, six other substitutions, and a unique C-terminal insertion of four to seven aa. The peculiar N-terminal domain of kinetoplastid eRF3, with prion-like features similar to those of its yeast homologs, which are known to promote translational readthrough (38), may have played a key role in the genetic code reassignment in the *Blastocrithidia* lineage, especially considering the substantial expansion of this domain in these trypanosomatids. Also notable is the *Blastocrithidia*-specific acidic region just downstream of the prion-like domain, which could affect eRF3 interaction with PABP and other termination factors (57). Although four *Blastocrithidia*-specific substitutions are present in the C-terminal helical domain of TrpRS1, which recognizes the tRNA^Trp^ anticodon as one of two primary identity determinants (59), we can only speculate whether Met/Gln290Ser may sense the uniquely shortened AS of tRNA^Trp^_CCA_ and whether other mutations may allosterically promote its accommodation into the anticodon binding pocket.

It is notable that within *Blastocrithidia,* there is no correlation of GC content in the coding regions and that in the 4fds, although it should be present assuming neutral evolution at these sites and considering that such a correlation is quite strong on a larger scale. Another unanticipated fact is that the proportion of ifRCs in conservative sites shows proper (i.e., negative) and strong correlation only for Glu codons (UAR/(UAR + GAR)), but not for Trp codons (UGA/(UGA + UGG)), where it is slightly positive (although not significant probably due to a low sample number). This leads to an unexpected conclusion that synonymous substitutions at the third codon position (both 4fds and the variable position in Trp codons belong to this category) are not neutral in *Blastocrithidia* and are governed by other unidentified factors, which seem to act differentially in different species of this genus. It is more surprising that under such circumstances, the synonymous substitutions at the first position of Glu codons appear to be perfectly neutral, although possessing such synonymity is an evolutionary novelty of *Blastocrithidia*.

The emergence of the *Blastocrithidia* lineage was accompanied by extensive gene loss, although this is not without precedent in trypanosomatids (60, 61). Of special interest is the loss of several RNA-interacting proteins and the eukaryotic translation initiation factor 3-associated factor eIF3j (62), which may have been either incompatible with, or rendered dispensable by, the non-canonical genetic code. Moreover, as expected, the ribonuclease PARN participating in the nonsense-mediated decay (NMD), a pathway generally responsible for degrading mRNAs carrying premature stop codons, was lost in *Blastocrithidia* spp. Although the main components of the NMD pathway (UPF1 and UPF2) are present in the genome of related *T. brucei* (63), their loss in the common ancestor after the genus *Trypanosoma* branched off (11), very likely constituted a condition favorable for stop codon reassignment in the *Blastocrithidia* lineage.

Although the vast majority of 200 OGs gained at the *Blastocrithidia* node are not functionally annotated, the expansion of OGs containing evolutionarily related minichromosome maintenance (MCM) proteins, namely, MCM8 and MCM9 helicases that form a heteromeric complex involved in homologous recombination (HR)-mediated DNA double-strand break repair in eukaryotes (64), is worth attention. It may indicate the reliance of *Blastocrithidia* on the HR-mediated double-strand break repair mechanism in the notable absence of the Ku70 and Ku80 proteins of the classical non-homologous end joining pathway (65).

The evolution of Euglenozoa in general, and Trypanosomatidae in particular, entails extensive remodeling of surface proteins, peptidases, kinases, and certain core metabolic enzymes (66, 67). Although we observed some alterations in the repertoire of these proteins at the ancestral node of *Blastocrithidia*, particularly noteworthy is the significant extent of changes in the repertoire of various proteins involved in nucleic acid metabolism (RNA-interacting proteins, transcription and translation factors, and ribosomal proteins). We assume that at least some of these changes might be connected to the adoption of the non-canonical genetic code of *Blastocrithidia* parasites. The ongoing transformation of their model representative, *B. nonstop*, into a genetically tractable organism provides a promise for functional studies shedding light on the highly improbable, yet increasingly better-documented alterations of the canonical genetic code.

## MATERIALS AND METHODS

### Origin and cultivation of studied species

For this work, we collected cultures of cyst-forming trypanosomatids of the genera *Blastocrithidia* and *Obscuromonas* (both from the subfamily Blastocrithidiinae). Among the selected species, there were two groups: (i) with a narrow known geographic range, such as *B. triatomae* (South America), *B. raabei* (Europe), *O. volfi* (Curaçao), *O. eliasi* (Papua New Guinea [PNG]), and *O. oborniki* (Africa and Madagascar) and (ii) widely distributed, namely, *B. nonstop* (Africa, Asia, Central and South America, Europe, and PNG), *B. frustrata* (Asia, Europe, and PNG), and *O. modryi* (Africa, South America, PNG, and Philippines) (17–19, 68, 69). Details concerning the origin of all studied isolates are provided in Table 1. All species were cultivated at 23°C in Schneider’s *Drosophila* medium (Thermo Fisher Scientific, Waltham, MA, USA) supplemented with 10% fetal bovine serum (Sigma-Aldrich/Merck, St. Louis, MO, USA), 100 µg/mL streptomycin, and 100 U/mL penicillin (both Thermo Fisher Scientific). Species identity was validated as described previously (70).

For northern blotting, *B. nonstop* was grown according to conditions described previously (11). *B. raabei* was grown at 25°C in flat flasks in RPMI 1640 and Schneider’s *Drosophila* medium (both Sigma-Aldrich/Merck, Darmstadt, Germany) mixed in 1:1 ratio and supplemented with 20% heat-inactivated fetal bovine serum, 100 U/mL penicillin, and 100 µg/mL streptomycin (all Biowest, Nuaillé, France). The procyclic form of *T. brucei* Lister 427 29–13 was cultured at 27°C in SDM79 medium (Sigma-Aldrich/Merck) containing 10% heat-inactivated fetal calf serum (Biowest), 5 µg/mL hemin (Sigma-Aldrich/Merck), 25 µg/mL hygromycin, and 10 µg/mL neomycin (both Sigma-Aldrich/Merck).

### Northern blotting

Total RNA from *B. raabei*, *B. nonstop*, and *T. brucei* was isolated with TRI Reagent using the manufacturer’s protocol. Ten micrograms of total RNA were separated on denaturing 8% PAGE with 8 M urea and electroblotted to Zeta-probe membranes (Bio-Rad Laboratories, Hercules, CA, USA), which were subsequently probed with ^32^P-labeled oligonucleotides (5'-gtcgcctgggttaaagccaga-3') specific for tRNA^Glu^_UUA_ as described previously (13). Images were taken with a Storm PhosphoImager (Molecular Dynamics/GE Healthcare, Chicago, IL, USA).

### Nucleic acid isolation and sequencing

Total DNA and RNA were isolated from 3 × 10^7^ to 1 × 10^8^ cells using the conventional phenol-chloroform method (71) and TRI reagent (Molecular Research Center, Cincinnati, OH, USA), respectively. DNA and RNA libraries were prepared and sequenced using the Illumina NovaSeq 6000 platform at Macrogen Europe (Amsterdam, Netherlands).

### Genome assembly

Raw DNA-Seq reads were adapter and quality-trimmed using BBDuk v38.98 (72) keeping all reads or those with a minimum length of 75 nt. Reads were error corrected and assembled in three strategies: (i) all reads were error corrected (i.e., using the --careful option) and assembled by SPAdes v3.13.0 (73); (ii) only reads with ≥75 nt were error corrected and assembled by SPAdes; and (iii) only reads with ≥75 nt were error corrected by Karect (74) and then assembled by SPAdes without error correction. QUAST v5.2.0 (75) was run on the assembled contigs from all three strategies, and the assembly with the best statistics (N50, length of the largest contig, number of contigs above 500 nt, number of contigs above 50,000 nt) (Table S1A) was chosen. Contigs assembled with strategy iii showed the best statistics for all species, except for *O. volfi*, which had better values for contigs assembled with strategy i. Scaffolding was done using Platanus v1.2.4 (76) in two rounds intercalated with GapCloser v1.12 from SOAPdenovo2 for gap filling (77).

Identities of each species were re-assessed by extracting small subunit rRNA gene sequences (18S rDNAs). This identified a single kinetoplastid 18S rDNA in each assembly. Potential contamination was further assessed by BlobTools v1.0 (78). The scaffolds shorter than 500 nt and those showing nucleotide identity over 95% and query coverage over 85% to non-euglenozoan sequences in BLASTN v2.5.0+ searches (79) against the NCBI nucleotide database (download date: 8 May 2022) were removed. Scaffolds with non-euglenozoan hits below the specified thresholds were further screened by DIAMOND v2.0.15 (80) in sensitive mode against the NCBI non-redundant database (download date: 14 June 2022) and removed if non-euglenozoan sequences were retrieved as best hits. The decontaminated assemblies were submitted to Repeat-Modeler v2.0.4 (81) using the LTRStruct parameter. RepeatMasker v4.1.4 (82) with sensitive slow search was used for the identification of repeats and soft-masking using the database built with RepeatModeler. The completeness of the final assemblies was evaluated by BUSCO v5 (83) in protein mode using eukaryota_odb10 and euglenozoa_odb10 reference databases.

### Transcriptome assembly

Raw RNA-Seq reads were adapter and quality-trimmed using BBDuk v38.98 keeping reads with a minimum length of 50 nt. Trimmed reads were *de novo* assembled using Cufflinks v2.2.1 (84) with default parameters.

### Genuine stop codon identification

To identify genuine stop codons, TBLASTN searches (-e-value 1E-20; -max_target_seqs 1) using *B. nonstop* proteins as queries against the genome assemblies of other *Blastocrithidia* spp. were performed. Only alignments with complete 3′ ends of the query proteins were analyzed further. A custom Python script was used to identify a following codon in the *Blastocrithidia* genome sequence after the end of the alignment.

### Gene prediction and annotation

The genetic code of each species was assessed by Codetta v2.0 (85). Protein-coding genes of *Blastocrithidia* spp. were predicted based on evidence taken from transcriptomic read mapping, *trans*-splicing sites, and the alignments with the reference proteins from *B. nonstop* (11, 86) and trypanosomatid species available in TriTrypDB release 52 (87). Mapping of *trans*-splicing sites was performed by SLaP mapper (88) using a partial sequence of the *B. nonstop* spliced leader RNA (AGTTTCTGTACTTTATTG) with a minimal length of 6 nt. Trimmed RNA-Seq reads were mapped onto the genome assembly using HISAT2 v2.0.5 (89) and BEDtools v2.30.0 (90). All regions that had a minimum coverage of 10 and a BLASTX hit (-e-value 1E-05) in the NCBI non-redundant protein database were kept. These hits were extracted as proteins and added to the trypanosomatid query database. Reference protein alignments were generated using TBLASTN (-e-value 1E-10; -max_target_seqs 100). Where multiple high-scoring pairs (HSPs) were found, only those with identical strands and frames were stored for a given target/hit. For each query-target pair, minimum and maximum coordinates were recorded (from all stored HSPs), and then, the coordinates of the closest in-frame AUG and UAA to these BLAST-determined boundaries were identified (i.e., the conserved protein region). A more upstream AUG was discarded if a spliced leader site was found in the 5' homology region (i.e., in the range covered by the protein query); in that case, a downstream AUG was selected as the gene start. Following all candidate target range collection, overlapping target ranges were reduced to include only the longest range. Up to 10 nucleotide overlaps were allowed between partially overlapping gene models. Predicted protein sequences of *Blastocrithidia* spp. were annotated as for *B. nonstop* (86) but including *B. nonstop* in the reference data set.

The average GC% in ORFs was 37%–39%. A preliminary analysis of sequences upstream and downstream of the assumed genuine stop codon showed that in each *Blastocrithidia* species, 79%–81% of sequences had at least one additional putative stop codon within 30 codons from the genuine stop codon (i.e., “filtered data set”). The GC% of these regions (average over 120 downstream nucleotides) dropped to an average of 23%–26%, consistent with the intergenic GC% and a relaxation of the 3GC bias. Stop codon and nucleotide frequencies analyses were performed with the full and filtered data set to eliminate bias caused by gene model errors.

Uncultured *Blastocrithidia* sp. ex *Lygus hesperus* represents contamination of the transcriptomic data of *L. hesperus* (NCBI BioProject ID: PRJNA238835) (10). Sequences of the trypanosomatid were identified by BLASTX searches (-e-value 1E-05; -max_target_seqs 1) against predicted proteins of *B. nonstop* and *B. frustrata*. The identified hits were then translated in the corresponding open reading frame with the *Blastocrithidia* genetic code. Protein-coding genes of *Obscuromonas* spp. were predicted and annotated by Companion Protozoa v1.0.2 (91). Predicted proteins shorter than 30 aa were removed from the data sets. Proteins of specific interest were manually checked, and their predicted sequence was adjusted when necessary.

Secondary structures of proteins were *de novo* predicted by AlphaFold2 (92) integrated in the ColabFold v1.5.5 (93) notebooks or AlphaFold3 (94) and were visualized and overlaid in ChimeraX v1.9 (95). Protein domains were annotated using InterProScan v5.55-88.0 (96) and the Pfam database (97). Functional annotation and clusters of orthologous genes (COG) functional category assignment were performed by eggNOG-mapper v2.1.10 (98). For statistical purposes, proteins falling within two or more COG categories were counted multiple times (per each category).

Genes encoding rRNAs were identified by TBLASTN v2.9.0+ searches using the *B. nonstop* rDNA sequences as queries. Genes encoding tRNAs were predicted by tRNAscan-SE v2.0.11 (99) and ARAGORN v1.2.38 (100). The full tRNA^Glu^_UUA_ sequence of *B. raabei* was reconstructed by BLASTN searches against the raw reads and using the partial tRNA^Glu^_UUA_ sequence as a query.

### Ploidy analysis

As previously described (15), for each scaffold, mean read depths were calculated in successive non-overlapping 1 kb windows using Mosdepth v.0.3.3 (101) in default settings and then served to obtain a median-of-means (MOM) estimate. For each species, the median genome coverage was calculated based on those of the 100 largest scaffolds. The ratio (R) between the scaffold’s MOM coverage and the median genome coverage was used to define somy: 0.25 ≥ R < 0.75 – monosomic; 0.75 ≥ R ≤ 1.25 – disomic; 1.26 > R ≤ 1.75 – trisomic; 1.76 > R ≤ 2.25 – tetrasomic; 2.26 > R– pentasomic or higher. The somy of each scaffold was inferred assuming that most of the scaffolds/chromosomes are in the disomic state (15).

### Codon usage

Coding sequences (CDSs) of predicted proteins were extracted from genomic assemblies using cdseq v1.0.1 (https://github.com/glarue/cdseq). For *B. nonstop*, CDSs of seven proteins encoded in the mitochondrial DNA (13) included in the original data set were removed. For *Obscuromonas* spp., CDSs of predicted pseudogenes and those split into ≥2 parts were excluded. Codon usage of each CDS was analyzed by a custom Python script. Mean codon usage for selected trypanosomatids (*Blechomonas ayalai* B08-376, *Bodo saltans* Lake Konstanz, *Crithidia fasciculata* Cf-Cl, *Leishmania major* Friedlin, *Paratrypanosoma confusum* CUL13, and *Trypanosoma brucei brucei* TREU927) was taken from VEuPathDB.

To avoid a large sample bias for COG comparisons, pairwise statistical comparisons were made on 40 randomly chosen genes using the two-tailed Mann-Whitney *U* test, adjusted for multiple comparisons by the Benjamini/Hochberg correction.

Relative adaptiveness of a codon (w) was calculated from the top 290 most highly expressed *B. nonstop* proteins as determined by mass spectrometry (i.e., proteins with >0.1 A.U.). The codon frequencies for the corresponding CDSs were calculated, and then, for each amino acid, the frequencies of codons were divided by the frequency of the most abundant codon in that group (102). The codon adaptation index (CAI) for a CDS was then calculated as the geometric mean of the w values (see equation 7 from reference 102).

Logo plots for motifs flanking the ifRCs and genuine stop codons were generated with the logomaker algorithm of Python (v0.8) using the counts-to-probability transformation and then normalized to background ORF nucleotide frequencies (i.e., enrichment). The statistical significance of nucleotide enrichment in the flanking region was assessed by performing a Bonferroni-corrected binomial test on 40 iterations of randomly sampled motifs (*n* = 200 per iteration), averaged to account for variability (Python scipy binomtest v1.15; statsmodels multipletests v0.14.4). Sample sizes for the random selection were estimated using Cohen’s h effect size for proportions and a two-tailed Z-test approximation for binomial proportions, assuming a statistical power of 0.9 and a 10% difference between background and enriched nucleotide frequencies.

### Homology searches

Proteins of the Sec utilization toolkit and selenoproteins previously identified in kinetoplastids (31, 103) served as queries in BLASTP and TBLASTN searches (-e-value 1E-05) against Blastocrithidiinae predicted proteomes and genomes, respectively. The Selenoprofiles v4.4.9 tool (104) was used to identify homologs of selenoproteins from other eukaryotes in *Obscuromonas* genomes, and these in turn served as queries in BLAST searches in *Blastocrithidia* data sets as above. TrpRS, eRF1, eRF3, PABP1, and PABP2 were identified by BLAST searches as above. Prion-like domains of eRF3 proteins were identified and visualized by the PLAAC tool (105).

### Phylogenomic analysis and divergence times estimation

For the phylogenomic analysis, predicted reference proteomes missing in the original PhyloFisher database v1.0 (20) were obtained from TriTrypDB release 61 (*Angomonas deanei* Cavalho ATCC PRA-265, *Blechomonas ayalai* B08-376, *Crithidia fasciculata* Cf-Cl, *Endotrypanum monterogeii* LV88, *Leishmania braziliensis* MHOM/BR/75 /M2904, *Leishmania martiniquensis* LEM2494, *Leishmania mexicana* MHOM/GT/2001 /U1103, *Porcisia hertigi* MCOE/PA/1965 /C119, *Trypanosoma congolense* IL3000, *Trypanosoma cruzi* CL Brener Esmeraldo-like, and *Trypanosoma vivax* Y486), NCBI GenBank (*B. nonstop* P57 GCA_028554745.1, *Trypanoplasma borreli* Tt-JH PRJNA549827, *Phytomonas* sp. EM1 GCA_000582765.1, *Phytomonas* sp. Hart1 GCA_000982615.1, and *Perkinsela* sp. CCAP1560 GCA_001235845.1), and this study (*Blastocrithidia* spp., *Obscuromonas* spp.; see “Gene prediction and annotation”). A standard database enrichment pipeline was performed with PhyloFisher v1.2.13 (20, 106). The resulting multi-protein alignment was used as an input for phylogeny inference by IQ-TREE v2.3.5 (with the ELM + C60 + G model for guide tree inference, followed by PMSF analysis using the same model and the guide tree input with 1,000 replicates for ultrafast bootstraps (107) and a maximum of 5,000 iterations). The resulting phylogeny was perfectly congruent with a previous analysis (20).

Divergence times were calculated using the RelTime method (21) with a subset phylogenomic alignment and ultrametric subset phylogeny used as input. Specifically, Euglenida, Diplonemida, Kinetoplastida, and *Naegleria* (as outgroup) sequences and branches were extracted, and the ultrametric tree was calculated from the corresponding IQ-TREE subtree using r8s (108). The extracted alignment (with the above subset of species) was further trimmed with trimAl v.1.2rev59 (-gt 0.5) (109) and PhyloFisher’s fast_site_remover function (20) to remove gaps and positions with fast-evolving sites, respectively (22,612 positions remained). The timetree was computed with three calibration constraints, that is, Leishmaniinae: 120 MYA, sigma = 4; Euglenida: 450 MYA, sigma = 14; normal distribution (110, 111); and Euglenozoa root in three settings (1,000, 1,300, and 1,600 MYA, i.e., the minimum 95% CI, mean, and maximum 95% CI values in reference 23), since this strongly affected the tree age in the absence of a calibration point outside Euglenozoa. We used the WAG substitution model with invariant sites, local clock, and three gamma categories.

Sequence identity and its distribution (kernel density) were calculated from a subset of coding sequences that were determined as reciprocal best BLAST hits and aligned by Muscle5 (112).

### Influence of GC content on ifRC usage and their changes in the evolution of *Blastocrithidia*

This analysis was performed for all five species with genomic and/or transcriptomic data available (i.e., *B. nonstop, B. triatomae, B. raabei, B. frustata*, and *Blastocrithidia* sp. ex *L. hesperus*) with the four species of *Obscuromonas* used as the closest reference. To this end, we sampled all alignment columns from the phylogenomic data set with Glu and Trp conserved across *Blastocrithidia* and *Obscuromonas* and lacking gaps or missing data (1,066 and 202 positions, respectively). For each of the two amino acids, the proportions of ifRCs, that is, UAR/(UAR + GAR) and UGA/(UGA + UGG), were estimated. Such an approach allowed us to estimate the dynamics of these codons not depending on the selection due to factors other than GC content. In addition, we analyzed 4-fold degenerated sites, which are known to evolve neutrally (113), and, therefore, their GC content should be theoretically determined only by that of ORFs. The obtained values were mapped to a cladogram depicting the phylogenetic relationships as inferred in the phylogenomic analysis (Fig. 1) and used for correlation analyses.

### Gene family evolution

Predicted proteins of all *Blastocrithidia* and *Obscuromonas* spp. produced in this study, *B. nonstop* (11), *B. triatomae* (16), *O. modryi* (15), and 13 reference kinetoplastids (*Leishmania major* Friedlin, *Leishmania mexicana* MHOM/GT/2001 /U1103, *Leishmania martiniquensis* LEM2494, *Porcisia hertigi* MCOE/PA/1965 /C119, *Endotrypanum monterogeii* LV88, *Leptomonas pyrrhocoris* H10, *Crithidia fasciculata* Cf-Cl, *Angomonas deanei* Cavalho ATCC PRA-265, *Blechomonas ayalai* B08-376, *Trypanosoma brucei brucei* TREU927, *Trypanosoma cruzi* CL Brener Esmeraldo-like, *Paratrypanosoma confusum* CUL13, and *Bodo saltans* Lake Konstanz) obtained from the TriTrypDB release 61 were clustered to orthologous groups using OrthoFinder v2.0.0 (114) under default settings. Count v10.04 (115) was employed to analyze gene gains and losses, as well as gene family expansions and contractions with Dollo and Wagner (gain penalty set to 3) parsimony algorithms, respectively. KEGG IDs assignment and pathway mapping were done using BlastKOALA (116).

## Data Availability

Raw DNA and RNA sequencing reads, and genome and transcriptome assemblies of species sequenced in this study were deposited under BioProject PRJNA1191792. Annotated protein data sets are available from Figshare under the link https://figshare.com/projects/Blastocrithidiinae_predicted_proteomes/230816. Any additional information is available from the corresponding authors.

## SUPPLEMENTAL MATERIAL

Supplemental Figures mbio.00885-25-s0001.pdf Figures S1 to S16.

Supplemental Tables mbio.00885-25-s0002.xlsx Tables S1 to S12.

ASM does not own the copyrights to Supplemental Material that may be linked to, or accessed through, an article. The authors have granted ASM a non-exclusive, world-wide license to publish the Supplemental Material files. Please contact the corresponding author directly for reuse.
