# Supplementary material for: Comparative genomic analysis of trypanosomatid protists illuminates an extensive change in the nuclear genetic code
Source: mBio. 2025 Apr 28;16(6):e00885-25. doi: 10.1128/mbio.00885-25 (PMC12153298; doi:10.1128/mbio.00885-25)
Supplement: Supplemental Figures — Figures S1 to S16. [file mbio.00885-25-s0001.pdf]

Comparative genomic analysis of trypanosomatid protists illuminates an extensive change in the nuclear genetic code

Kristína Záhonová[1,2,3,4,#], Zoltán Füssy[5,6,#], Amanda T. S. Albanaz[1,$], Anzhelika Butenko[1,2,6], Ambar Kachale[1,6,$], Natalya Kraeva[1], Arnau Galan[1], Alexandra Zakharova[1], Bojana Stojanova[1,$], Jan Votýpka[2,3], Alexei Y. Kostygov[1,7], Viktoria V. Spodareva[1,7], Marina N. Malysheva[7], Alexander O. Frolov[7], Igor B. Rogozin[1], Zdeněk Paris[1,6], Leoš Shivaya Valášek[8], Vyacheslav Yurchenko[1,*], Julius Lukeš[2,6,*]

[1] Life Science Research Centre, Faculty of Science, University of Ostrava, Ostrava, Czechia

[2] Institute of Parasitology, Biology Centre, Czech Academy of Sciences, České Budějovice (Budweis), Czechia

[3] Department of Parasitology, Faculty of Science, Charles University, BIOCEV, Vestec, Czechia

[4] Division of Infectious Diseases, Department of Medicine, Faculty of Medicine and Dentistry, University of Alberta, Edmonton, Canada

[5] Scripps Institution of Oceanography, University of California San Diego, La Jolla, USA

[6] Faculty of Science, University of South Bohemia, České Budějovice (Budweis), Czechia

[7] Zoological Institute, Russian Academy of Sciences, St. Petersburg, Russia

[8] Institute of Microbiology, Czech Academy of Sciences, Prague, Czechia

[#] These authors contributed equally to this work

[$] Current addresses: Instituto de Ensino e Pesquisa Santa Casa, Belo Horizonte, Brazil (A.T.S.A.); Harvard University, Cambridge, USA (A.K.); Faculty of Science, Masaryk University, Brno, Czechia and University Federico II, Naples, Italy (B.S.)

[*] **Corresponding authors:** vyacheslav.yurchenko@osu.cz (V.Y.); jula@paru.cas.cz (J.L.)

SUPPLEMENTARY FIGURES

Fig. S1. Phylogenetic tree of eukaryotes inferred by PhyloFisher. The underlying alignment contained 323 species, 240 genes, and 70,922 positions. The maximum-likelihood phylogenetic tree was inferred by IQ-TREE v2.3.5 with the ELM+C60+G model for guide tree, followed by PMSF analysis using the same model and the guide tree input with 1,000 replicates for ultrafast bootstraps and a maximum of 5,000 iterations. All support values were 100 except those that are shown. Organisms belonging to Blastocrithidiinae and sequenced in this work are in bold and underlined, respectively.

Fig. S2. Genetic code predictions from Codetta. Note that the results for species of the same genus were the same, and thus simplified here for visualization purposes. ifRCs in *Blastocrithidia* are in colors and their log decoding probabilities are shown above the table.

Fig. S3. Genome assembly completeness and partial duplication analysis. A) The completeness of genome-derived proteomes was assessed by BUSCO v5 using the odb10_euglenozoa (left) and odb10_eukaryota (right) databases. **B)** Length-normalized read mapping rates for scaffolds with only/no duplicated genes (left) or duplicated/single copy genes (right). Duplications determined by OrthoFinder clustering. In *B. triatomae*, similar read coverage of single-copy and duplicated genes and scaffolds suggests a *bona fide* partial genome duplication event. In *O. oborniki*, at least some gene duplications appear to be assembly artifacts, as the coverage of gene and scaffold duplicates is only 55% and 62%, respectively, relative to single-copy genes and scaffolds (dashed lines).

Fig. S4. Identification of $\mathbf{tRNA^{Glu}_{UUA}}$ in *B. raabei*. A) The full-length $tRNA^{Glu}_{UUA}$ was reconstructed by identifying reads by blastn searches that extended its sequence. **B)** Ten µg of total RNA was resolved by 8% urea-PAGE, followed by the northern blot analysis using ^{32}P-labeled probe specific for $tRNA^{Glu}_{UUA}$. *B. nonstop* and *T. brucei* served as positive and negative controls, respectively. Ethidium bromide (EtBr)-stained urea-PAGE served as loading control.

Fig. S5. Structural changes to *Blastocrithidia* TrpRS. Multiple alignment of kinetoplastid TrpRS sequences. Blue boxed region corresponds to the anticodon-binding domain (5). Positions differing in *Blastocrithidia* spp. and the majority of kinetoplastid sequences are marked by pink arrows.

Fig. S6. $\mathbf{tRNA^{Sec}_{UCA}}$ in Blastocrithidiinae recognizing in-frame UGA. The UCA anticodon is highlighted in pink. Differences in the tRNA sequences of *Blastocrithidia* and *Obscuromonas* spp. are shown with grey background.

Fig. S7. Selenoproteins in Blastocrithidiinae. Multiple sequence alignment of kinetoplastid selenoproteins SelK (**A**), SelT (**B**), and SelTryp (**C**). Sec positions are highlighted in blue. Identity and conservation as in Fig. S5A.

Fig. S8. SelTryp using UGA as both Trp and Sec. Multiple sequence alignment of *Blastocrithidia* SelTryp nucleotide sequences. Below each nucleotide sequence, a conceptual translation of that sequence is shown. The UGA Trp and Sec codons are highlighted in pink and blue, respectively.

Fig. S9. Codon usage in cytosolic and mitochondrial ribosomal proteins. Codon usage was calculated as a percentage of the given codon in the respective group of synonymous codons for *Blastocrithidia* spp. (**A**) and reference trypanosomatids (**B**).

Fig. S10. Reassigned codons in different protein functional categories in *Blastocrithidia* spp. A) Predicted proteins were categorized into clusters of orthologous groups of proteins (COGs), the number of ifRCs in each category was summarized, and their frequencies were calculated as follows: UAA% = UAA/(UAA+GAA)×100, UAG% = UAG/(UAG+GAG)×100, and UGA% = UGA/(UGA+UGG)×100. **B)** Significance of ifRC frequency across functional categories. Any combination of COG categories significantly different in their ifRC frequency is marked with a square. To avoid large sampling bias, the significance was tested on 40 randomly chosen genes with multiple-comparison adjusted Mann-Whitney *U* test (see Materials and Methods). The comparisons are categorized by the species and the ifRC. Note that categories W, Y, and R were omitted to avoid low sample bias (n=<20 proteins). COG categories: M, Cell wall/membrane/envelope biogenesis; N, Cell motility; O, Posttranslational modification, protein turnover, chaperones; T, Signal transduction mechanisms; U, Intracellular trafficking, secretion, and vesicular transport; V, Defense mechanisms; W, Extracellular structures; Y, Nuclear structure; Z, Cytoskeleton; A, RNA processing and modification; B, Chromatin structure and dynamics; J, Translation, ribosomal structure and biogenesis; K, Transcription; L, Replication, recombination and repair; C, Energy production and conversion; D, Cell cycle control, cell division, chromosome partitioning; E, aa transport and metabolism; F, Nucleotide transport and metabolism; G, Carbohydrate transport and metabolism; H, Coenzyme transport and metabolism; I, Lipid transport and metabolism; P, Inorganic ion transport and metabolism; Q, Secondary metabolites biosynthesis, transport and catabolism; R, General function prediction only; S, Function unknown; -, No annotation.

Fig. S11. Structural changes to *Blastocrithidia* eRF1. Multiple alignment of kinetoplastid eRF1 sequences (note that GBHO01008089.1 represents a sequence of *Blastocrithidia* sp. ex *Lygus hesperus*). The conserved motifs important for stop codon recognition by eRF1 (30, 31) are highlighted in blue. The highlighted residue Ser74 (Ser70 in human numbering) in light orange corresponds to the critical substitution in *B. nonstop* previously shown to potentiate UGA readthrough (11). Positions differing between sequences of *Blastocrithidia* spp. and other kinetoplastids are marked by pink arrows. *Blastocrithidia*-specific insertion at the C-terminus is highlighted in pink box. Identity and conservation as in Fig. S5.

Fig. S12. Unique features of *Blastocrithidia* eRF3. A) Multiple sequence alignment of *S. cerevisiae* (NP_010457), *S. pombe* (NP_588225), and kinetoplastid eRF3 proteins (note that GBHO01036714.1 represents a sequence of *Blastocrithidia* sp. ex *Lygus hesperus*). Positions highlighted in blue

correspond to the residues whose substitutions suppressed an in-frame UGA codon in yeast (35, 109). Positions differing in *Blastocrithidia* spp. and the majority of kinetoplastid and yeast sequences are marked by pink arrows. *Blastocrithidia*-specific acidic region following the extended N-terminus is highlighted in pink box. Identity and conservation as in Fig. S5. **B)** The prionic character of the N-termini of kinetoplastid eRF3 are similar to that of *S. cerevisiae* eRF3 known for the Gln/Asn rich prion-like N-terminal domain. The plots are the output of the PLAAC tool (95).

Fig. S13. Stop codons terminating translation in Blastocrithidiinae. The frequency of different genuine stop codons was calculated based on their occurrence in CDSs of predicted proteins with complete 3'-end (Table S8). The GC-content of CDSs was calculated as (G+C)/(A+U+G+C)×100.

Fig. S14. Tandem stop codon counts across functional categories. The number of UAA stop codons within four codons downstream of the true stop are plotted (excluding the true stop), showing no clear trend of tandem codon enrichment across COG categories (explained in the Fig. S9 legend). In case of category W, some species have a uniform number of tandem stop codons (*B. nonstop* – 0, n= 2 genes; *B. triatomae* – 0, n=3; *B. frustrata* – 1, n=2).

Fig. S15. GC content in coding regions, 4-fold degenerate sites, and percentages of ifRCs at conservative Glu and Trp sites: evolution and correlation. A) Changes during the evolution of *Blastocrithidia*. The schematic phylogenetic tree is based on Fig. S1. **B)** Correlation analyses. x-axis represents ORF GC % in all cases. The trend (dotted) line was generated by linear regression.

Fig. S16. Gene gains/losses and gene family expansions/contractions mapped onto the kinetoplastid schematic tree. Note that the schematic tree is based on the phylogenetic tree from Fig. S1. Gene gains and losses were analyzed by Dollo parsimony algorithm, while gene family expansions and contractions by Wagner parsimony algorithm. The numbers of genes gained/lost and gene families expanded/contracted at certain nodes and species are depicted using bar plots placed at the nodes and to the right of the tree, respectively. The node numbers are shown in circles. Note that the reconstruction of gene family history for *B. saltans* and the trypanosomatid ancestral node may be biased due to the dataset composition and their position on the tree, therefore the corresponding estimates are not shown.

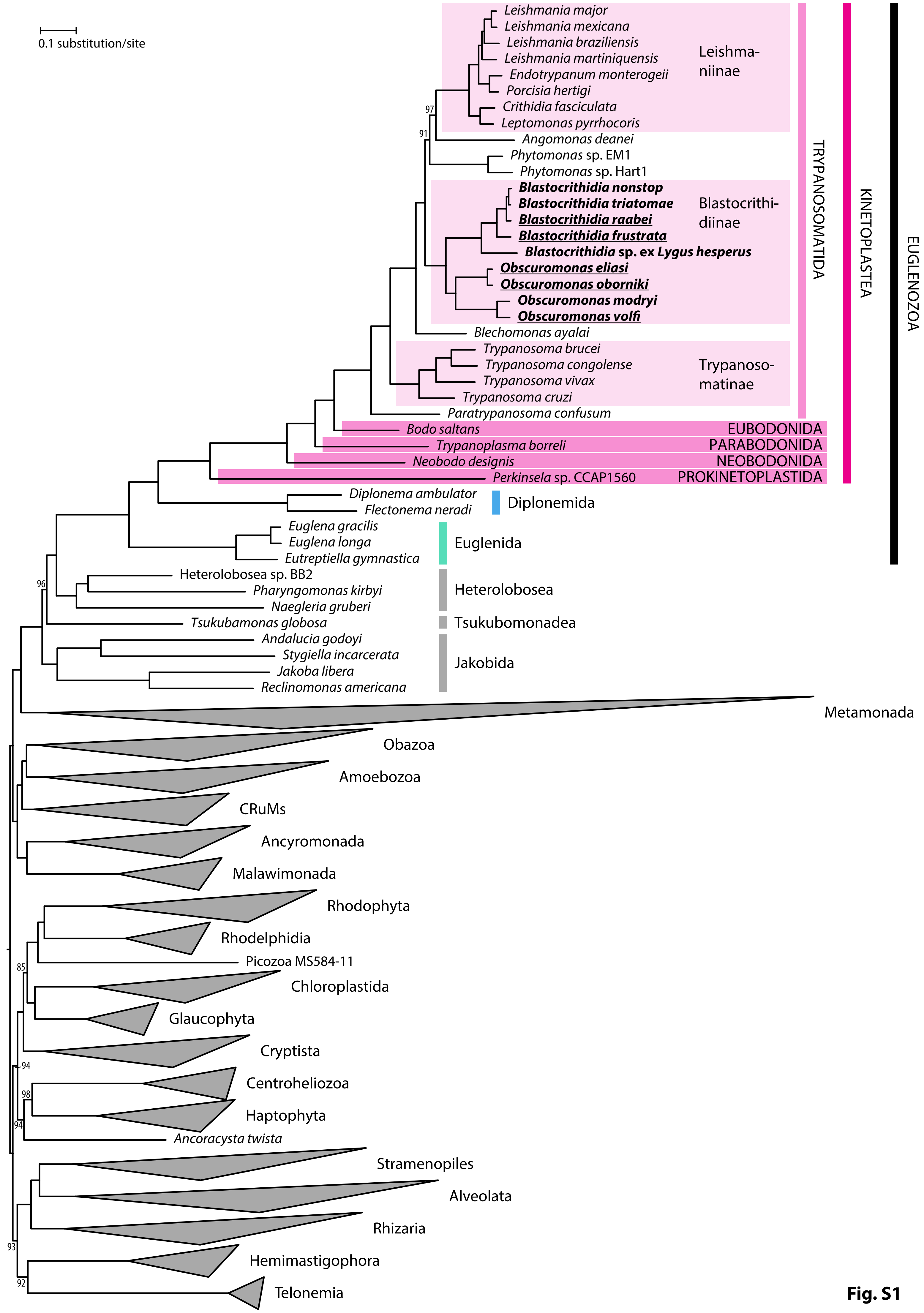
0.1 substitution/site
Leishmania major
Leishmania mexicana
Leishmania braziliensis
Leishmania martiniquensis
Endotrypanum monterogeii
Porcisia hertigi
Crithidia fasciculata
Leptomonas pyrrhocoris
Leishmaniinae
97
91
Angomonas deanei
Phytomonas sp. EM1
Phytomonas sp. Hart1
Blastocrithidia nonstop
Blastocrithidia triatomae
Blastocrithidia raabei
Blastocrithidia frustrata
Blastocrithidia sp. ex Lygus hesperus
Obscuromonas eliasi
Obscuromonas oborniki
Obscuromonas modryi
Obscuromonas volfi
Blastocrithidiinae
Blechomonas ayalai
Trypanosoma brucei
Trypanosoma congolense
Trypanosoma vivax
Trypanosoma cruzi
Trypanosomatinae
Paratrypanosoma confusum
TRYPANOSOMATIDA
Bodo saltans
EUBODONIDA
Trypanoplasma borreli
PARABODONIDA
Neobodo designis
NEOBODONIDA
Perkinsela sp. CCAP1560
PROKINETOPLASTIDA
KINETOPLASTEA
Diplonema ambulator
Flectonema neradi
Diplonemida
Euglena gracilis
Euglena longa
Eutreptiella gymnastica
Euglenida
EUGLENOZOA
Heterolobosea sp. BB2
Pharyngomonas kirbyi
Naegleria gruberi
Heterolobosea
96
Tsukubamonas globosa
Tsukubomonadea
Andalucia godoyi
Stygiella incarcerata
Jakoba libera
Reclinomonas americana
Jakobida
Metamonada
Obazoa
Amoebozoa
CRuMs
Ancyromonada
Malawimonada
Rhodophyta
Rhodelphidia
Picozoa MS584-11
85
Chloroplastida
Glaucophyta
Cryptista
94
Centroheliozoa
98
Haptophyta
94
Ancoracysta twista
Stramenopiles
Alveolata
Rhizaria
93
Hemimastigophora
92
Telonemia

Fig. S1

logP(E) = 0.0   logP(W) = 0.0

codon	TTT	TTC	TTA	TTG	TCT	TCC	TCA	TCG	TAT	TAC	TAA	TAG	TGT	TGC	TGA	TGG	CTT	CTC	CTA	CTG	CCT	CCC	CCA	CCG	CAT	CAC	CAA	CAG	CGT	CGC	CGA	CGG
**standard**	F	F	L	L	S	S	S	S	Y	Y	*	*	C	C	*	W	L	L	L	L	P	P	P	P	H	H	Q	Q	R	R	R	R
Blastocrithidia	F	F	L	L	S	S	S	S	Y	Y	E	E	C	C	W	W	L	L	L	L	P	P	P	P	H	H	Q	Q	R	R	R	R
Obscuromonas	F	F	L	L	S	S	S	S	Y	Y	*	*	C	C	*	W	L	L	L	L	P	P	P	P	H	H	Q	Q	R	R	R	R

codon	ATT	ATC	ATA	ATG	ACT	ACC	ACA	ACG	AAT	AAC	AAA	AAG	AGT	AGC	AGA	AGG	GTT	GTC	GTA	GTG	GCT	GCC	GCA	GCG	GAT	GAC	GAA	GAG	GGT	GGC	GGA	GGG
**standard**	I	I	I	M	T	T	T	T	N	N	K	K	S	S	R	R	V	V	V	V	A	A	A	A	D	D	E	E	G	G	G	G
Blastocrithidia	I	I	I	M	T	T	T	T	N	N	K	K	S	S	R	R	V	V	V	V	A	A	A	A	D	D	E	E	G	G	G	G
Obscuromonas	I	I	I	M	T	T	T	T	N	N	K	K	S	S	R	R	V	V	V	V	A	A	A	A	D	D	E	E	G	G	G	G

Fig. S2

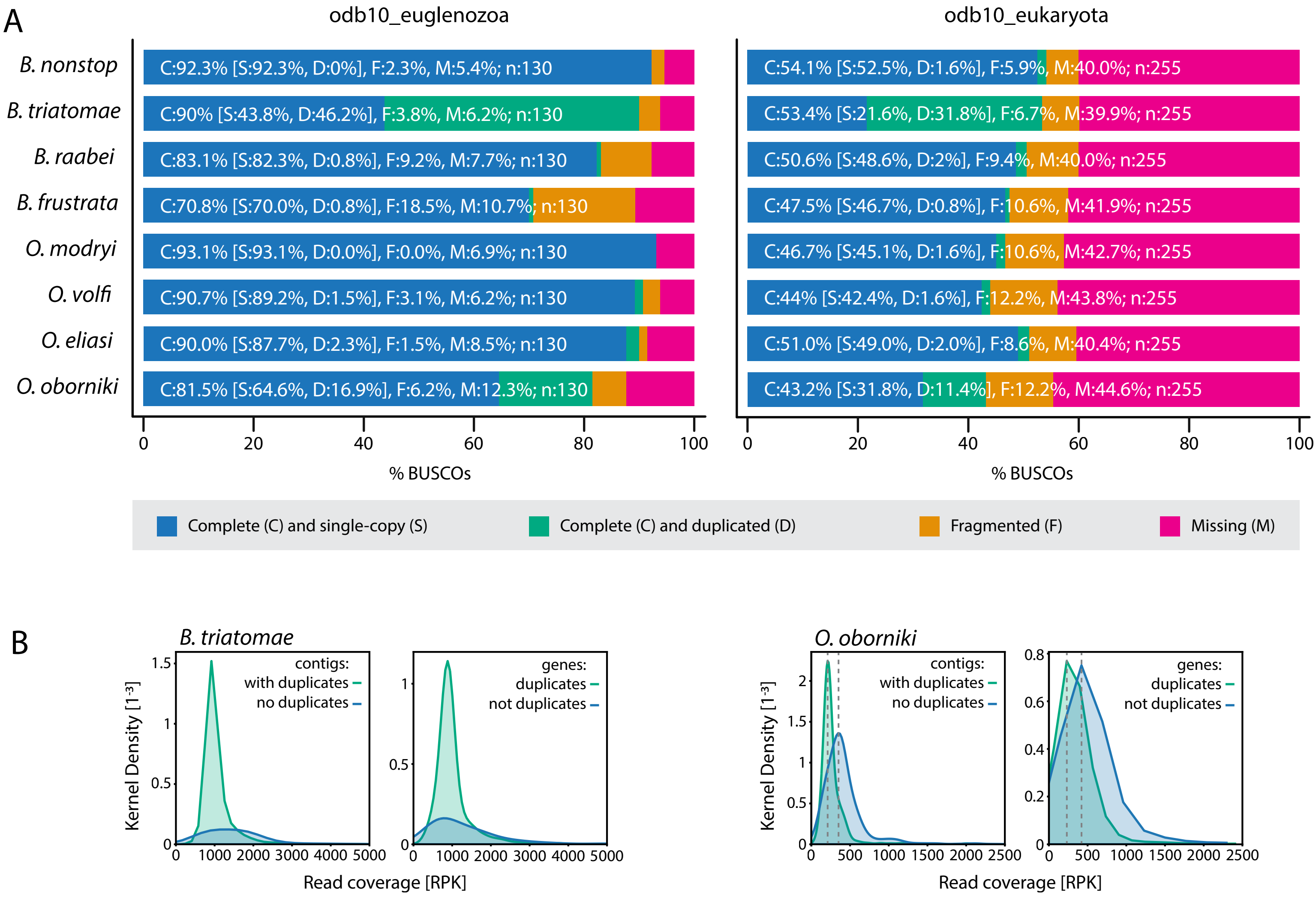

Fig. S3

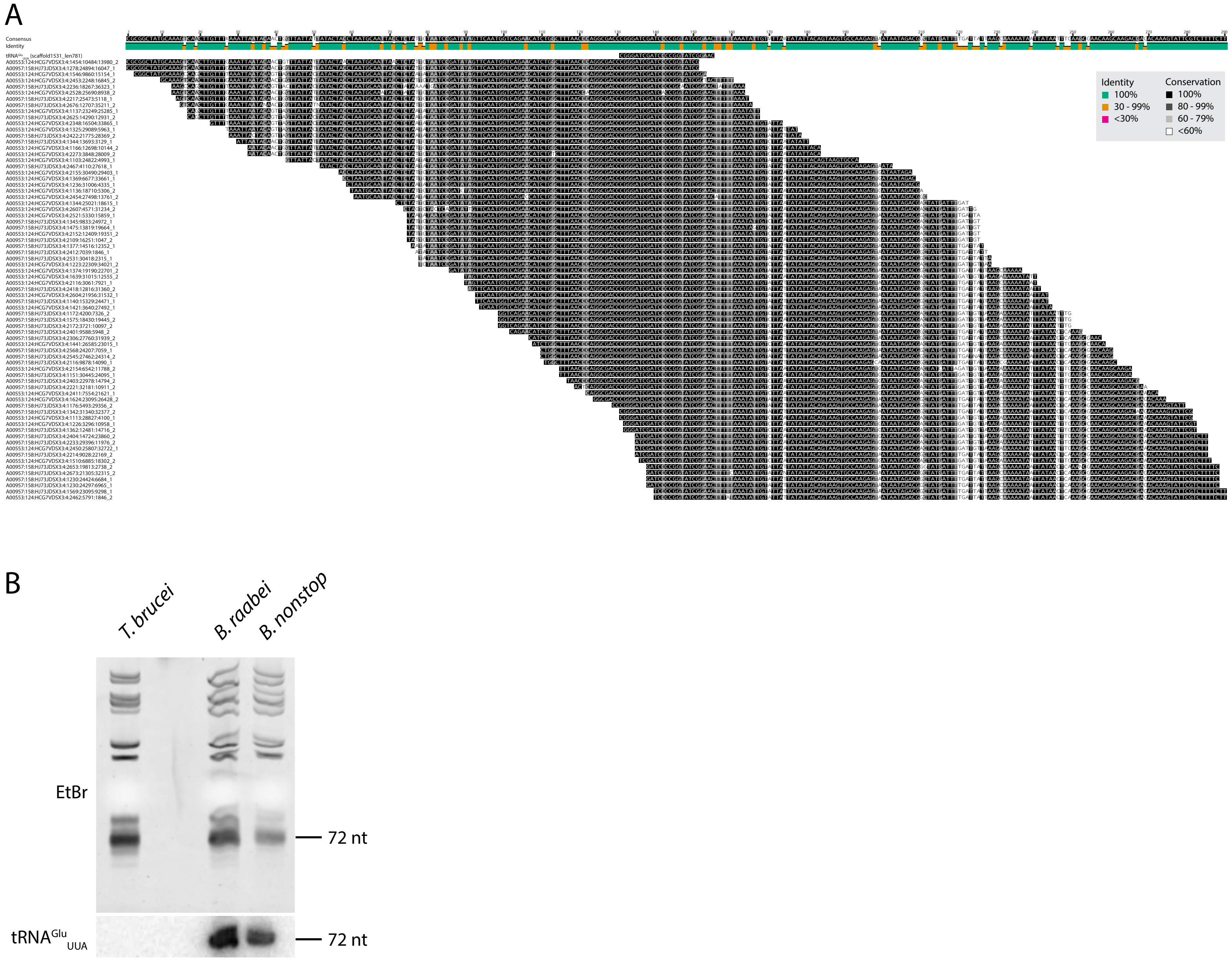
A
Consensus
Identity
Identity
100%
30 - 99%
<30%
Conservation
100%
80 - 99%
60 - 79%
<60%
B
T. brucei
B. raabei
B. nonstop
EtBr
72 nt
tRNA Glu UUA
72 nt

Fig. S4

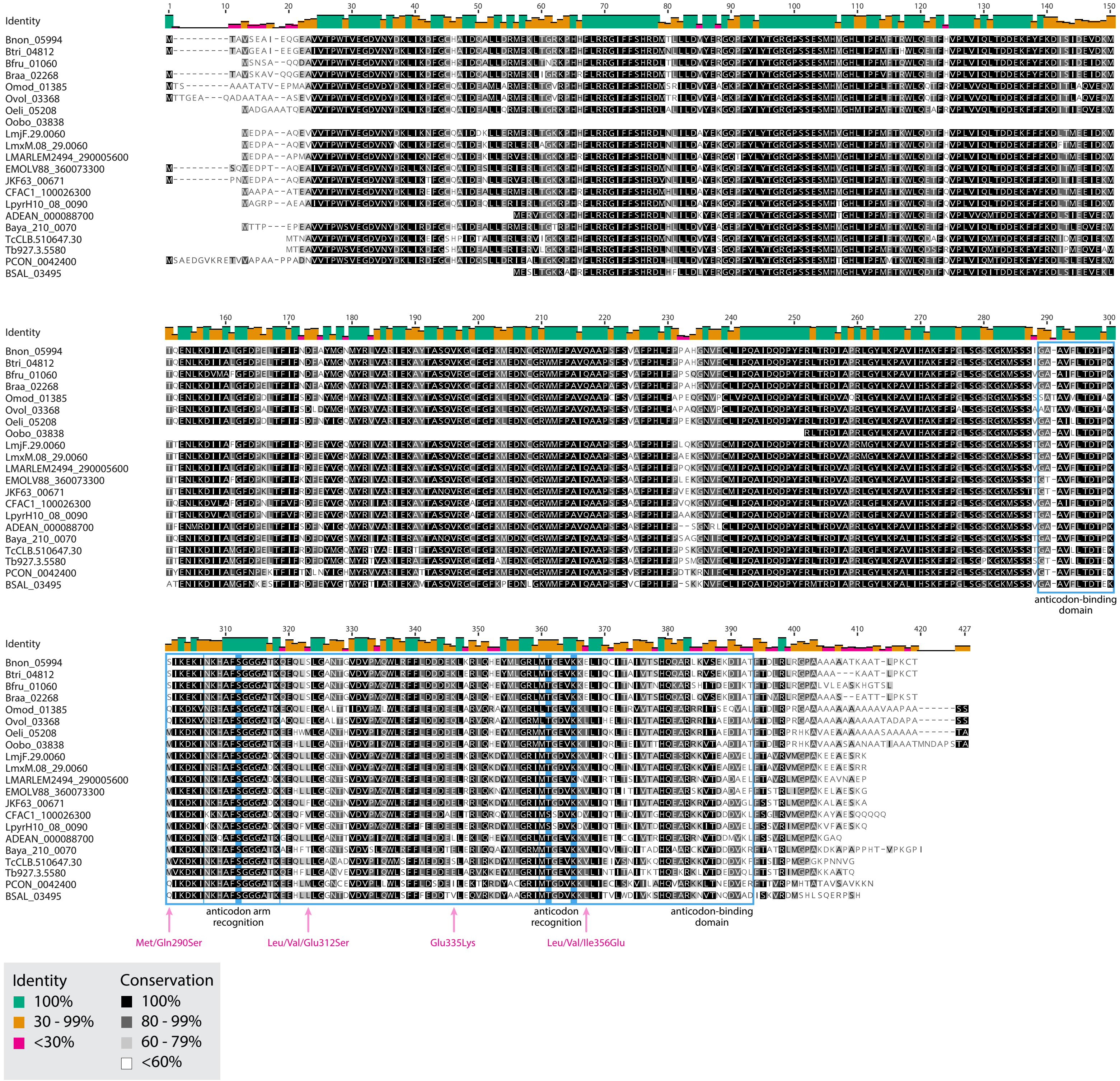
Identity
Bnon_05994
Btri_04812
Bfru_01060
Braa_02268
Omod_01385
Ovol_03368
Oeli_05208
Oobo_03838
LmjF.29.0060
LmxM.08_29.0060
LMARLEM2494_290005600
EMOLV88_360073300
JKF63_00671
CFAC1_100026300
LpyrH10_08_0090
ADEAN_000088700
Baya_210_0070
TcCLB.510647.30
Tb927.3.5580
PCON_0042400
BSAL_03495
anticodon-binding domain
anticodon arm recognition
anticodon recognition
anticodon-binding domain
Met/Gln290Ser
Leu/Val/Glu312Ser
Glu335Lys
Leu/Val/Ile356Glu
Identity
100%
30 - 99%
<30%
Conservation
100%
80 - 99%
60 - 79%
<60%

Fig. S5

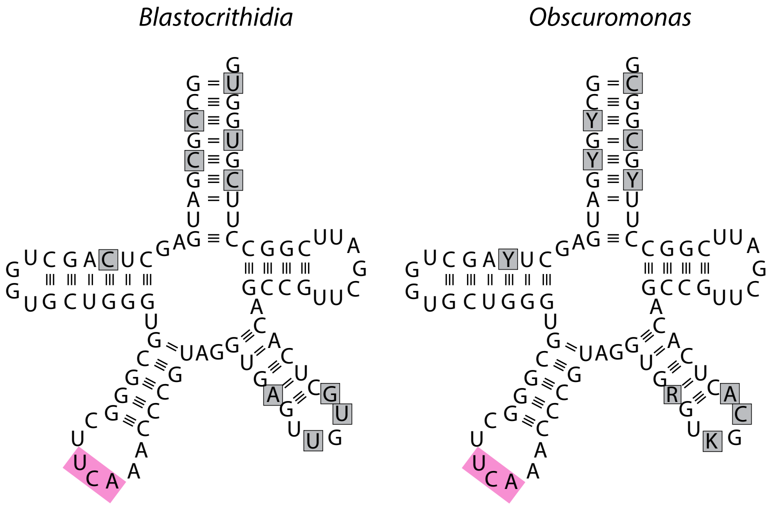
Blastocrithidia
Obscuromonas

Fig. S6

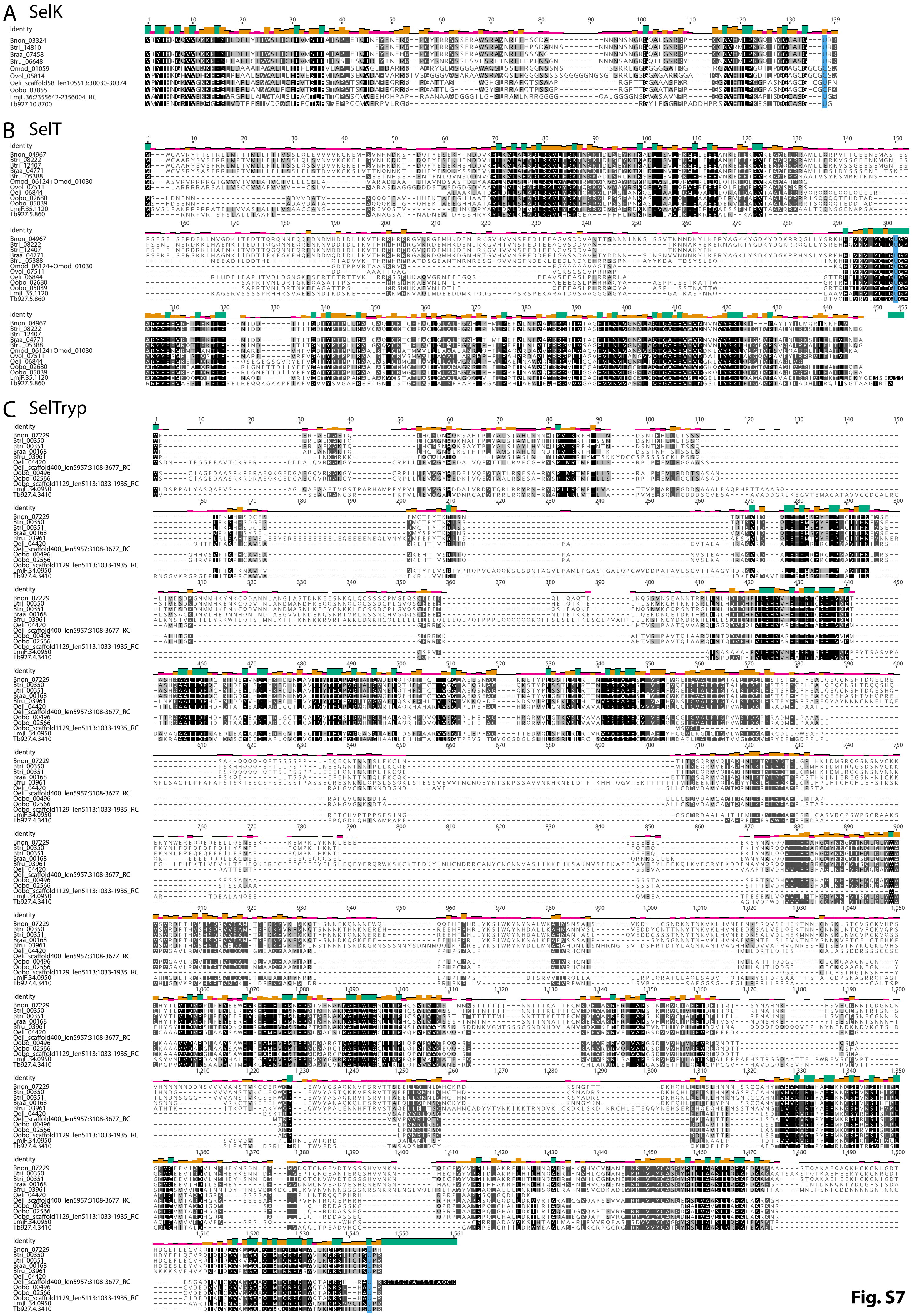
A SelK
Identity
Bnon_03324
Btri_14810
Braa_07458
Bfru_06648
Omod_01059
Ovol_05814
Oeli_scaffold58_len105513:30030-30374
Oobo_03855
LmjF.36:2355642-2356004_RC
Tb927.10.8700
B SelT
Bnon_04967
Btri_08222
Btri_12407
Braa_04771
Bfru_05388
Omod_06124+Omod_01030
Ovol_07511
Oeli_06844
Oobo_02680
Oobo_05039
LmjF.35.1120
Tb927.5.860
C SelTryp
Bnon_07229
Btri_00350
Btri_00351
Braa_00168
Bfru_03961
Oeli_04420
Oeli_scaffold400_len5957:3108-3677_RC
Oobo_00496
Oobo_02566
Oobo_scaffold1129_len5113:1033-1935_RC
LmjF.34.0950
Tb927.4.3410

Fig. S7

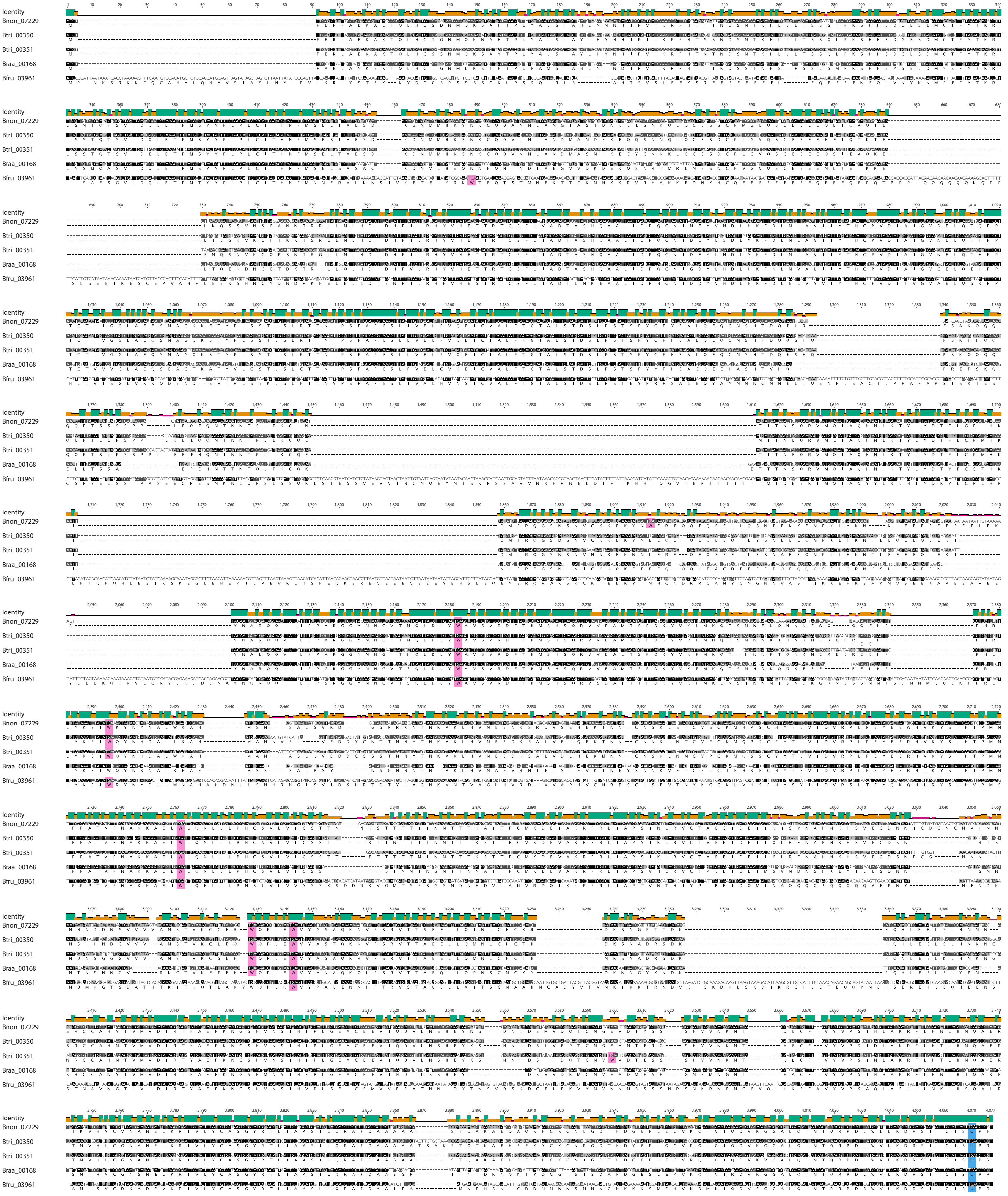
Identity
Bnon_07229
Btri_00350
Btri_00351
Braa_00168
Bfru_03961

Fig. S8

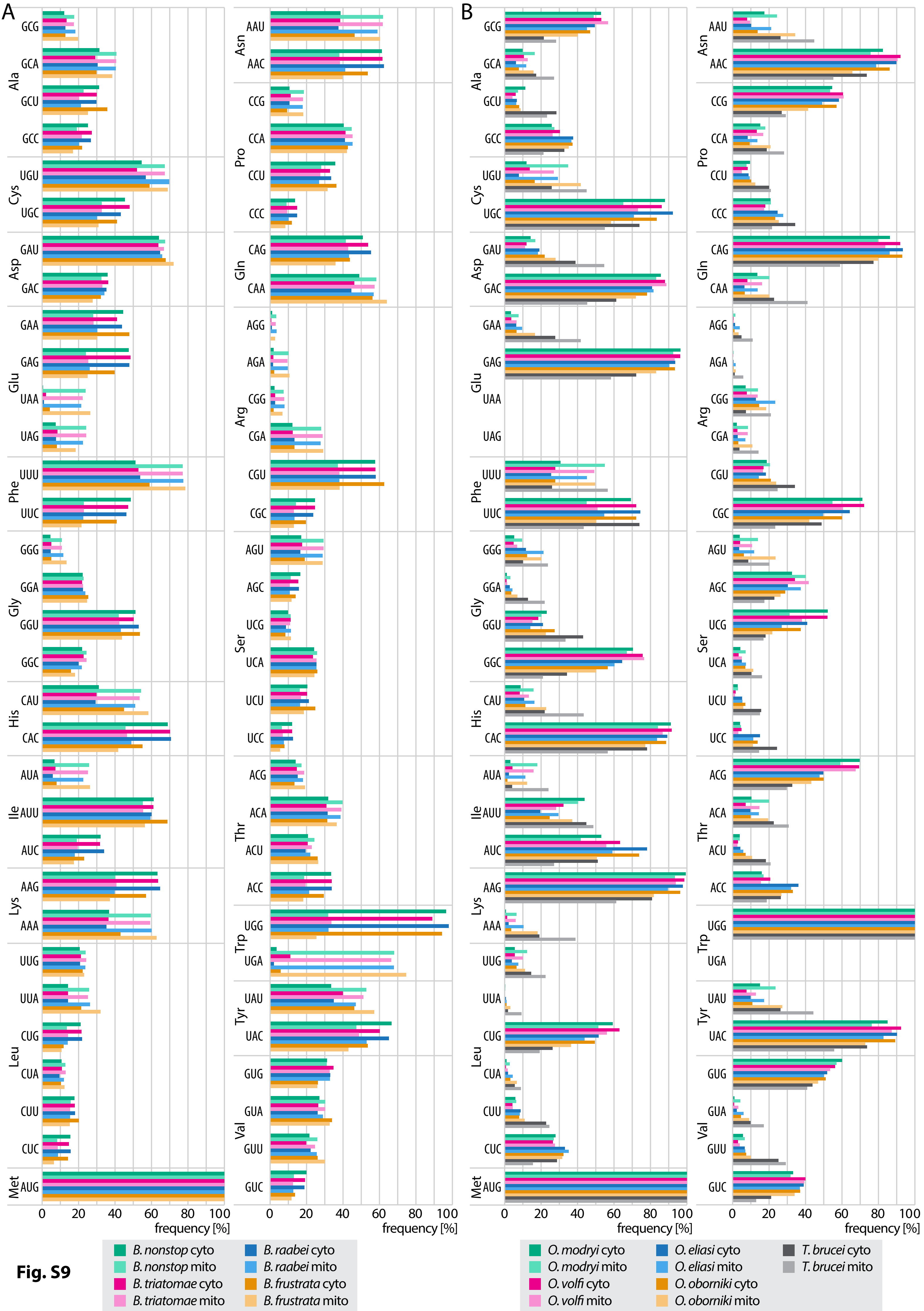

A
B
Ala
Cys
Asp
Glu
Phe
Gly
His
Ile
Lys
Leu
Met
Asn
Pro
Gln
Arg
Ser
Thr
Trp
Tyr
Val
frequency [%]
B. nonstop cyto
B. nonstop mito
B. triatomae cyto
B. triatomae mito
B. raabei cyto
B. raabei mito
B. frustrata cyto
B. frustrata mito
O. modryi cyto
O. modryi mito
O. volfi cyto
O. volfi mito
O. eliasi cyto
O. eliasi mito
O. oborniki cyto
O. oborniki mito
T. brucei cyto
T. brucei mito

Fig. S9

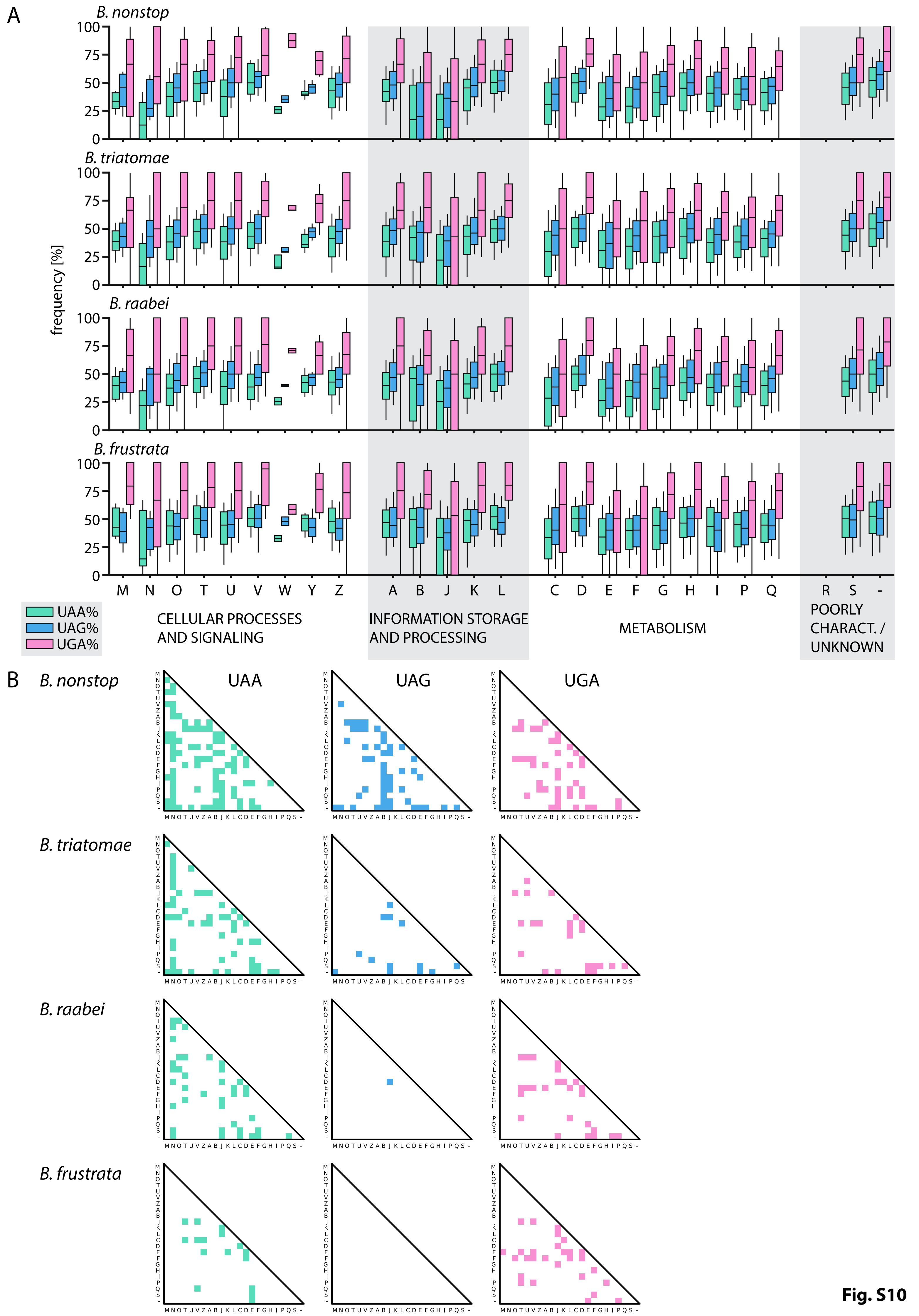
A
B. nonstop
B. triatomae
B. raabei
B. frustrata
frequency [%]
100
75
50
25
0
M N O T U V W Y Z A B J K L C D E F G H I P Q R S -
UAA%
UAG%
UGA%
CELLULAR PROCESSES AND SIGNALING
INFORMATION STORAGE AND PROCESSING
METABOLISM
POORLY CHARACT. / UNKNOWN
B
UAA
UAG
UGA

Fig. S10

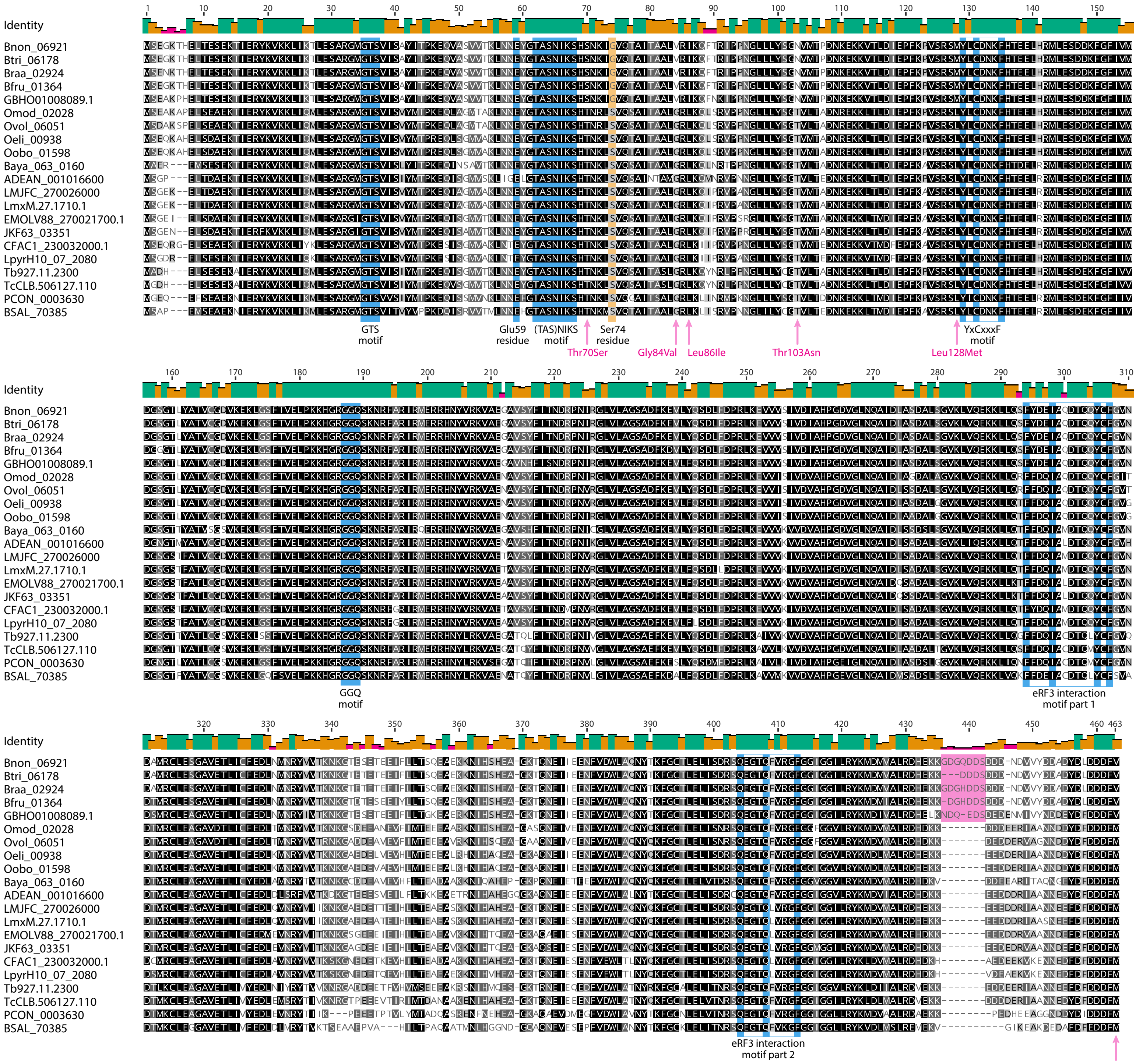
Identity
Bnon_06921
Btri_06178
Braa_02924
Bfru_01364
GBHO01008089.1
Omod_02028
Ovol_06051
Oeli_00938
Oobo_01598
Baya_063_0160
ADEAN_001016600
LMJFC_270026000
LmxM.27.1710.1
EMOLV88_270021700.1
JKF63_03351
CFAC1_230032000.1
LpyrH10_07_2080
Tb927.11.2300
TcCLB.506127.110
PCON_0003630
BSAL_70385
GTS motif
Glu59 residue
(TAS)NIKS motif
Ser74 residue
YxCxxxF motif
Thr70Ser
Gly84Val
Leu86Ile
Thr103Asn
Leu128Met
GGQ motif
eRF3 interaction motif part 1
eRF3 interaction motif part 2
Met426Val

Fig. S11

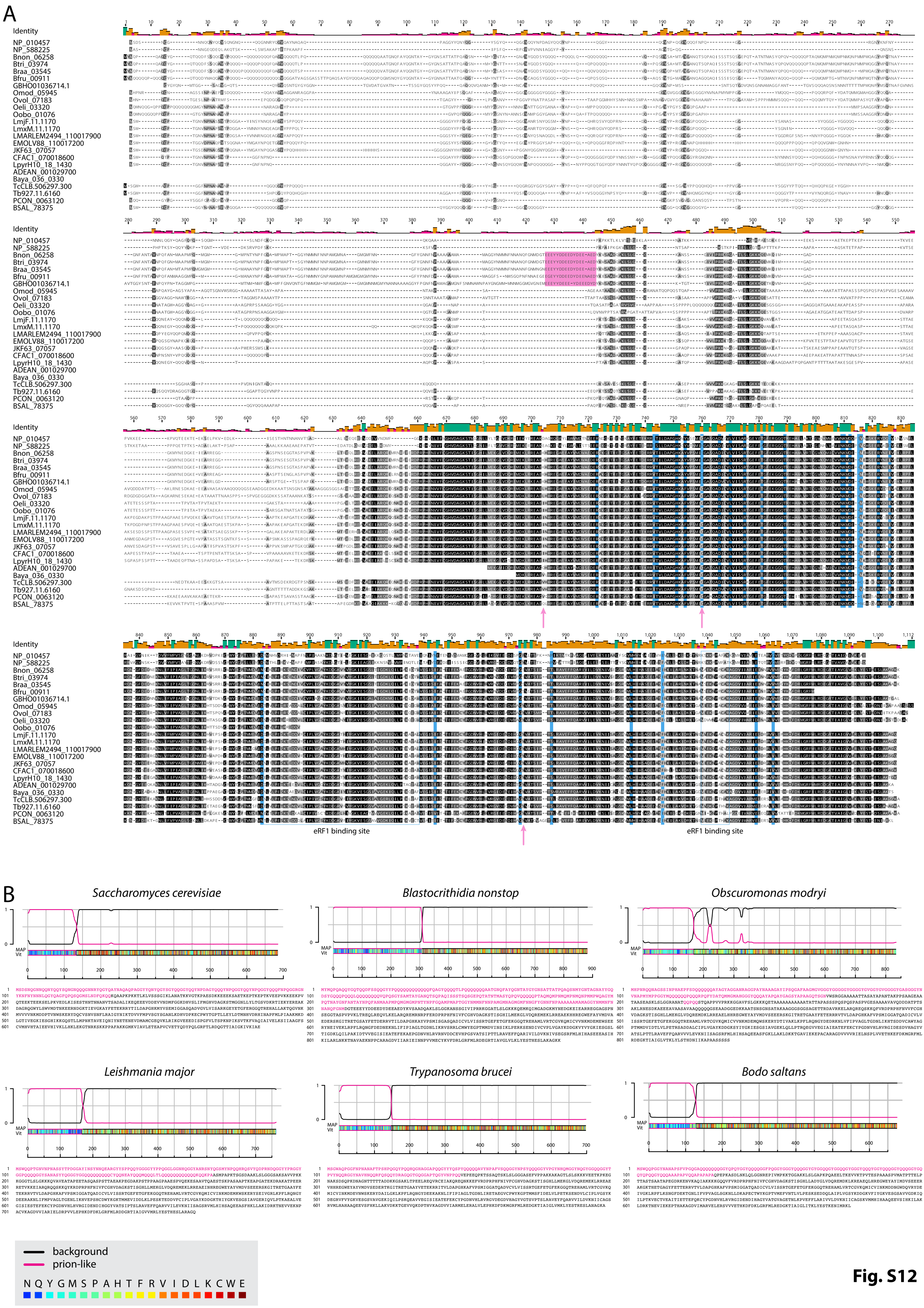
A
Identity
NP_010457
NP_588225
Bnon_06258
Btri_03974
Braa_03545
Bfru_00911
GBHO01036714.1
Omod_05945
Ovol_07183
Oeli_03320
Oobo_01076
LmjF.11.1170
LmxM.11.1170
LMARLEM2494_110017900
EMOLV88_110017200
JKF63_07057
CFAC1_070018600
LpyrH10_18_1430
ADEAN_001029700
Baya_036_0330
TcCLB.506297.300
Tb927.11.6160
PCON_0063120
BSAL_78375
eRF1 binding site
eRF1 binding site
B
Saccharomyces cerevisiae
Blastocrithidia nonstop
Obscuromonas modryi
Leishmania major
Trypanosoma brucei
Bodo saltans
MAP
Vit
background
prion-like
N Q Y G M S P A H T F R V I D L K C W E

Fig. S12

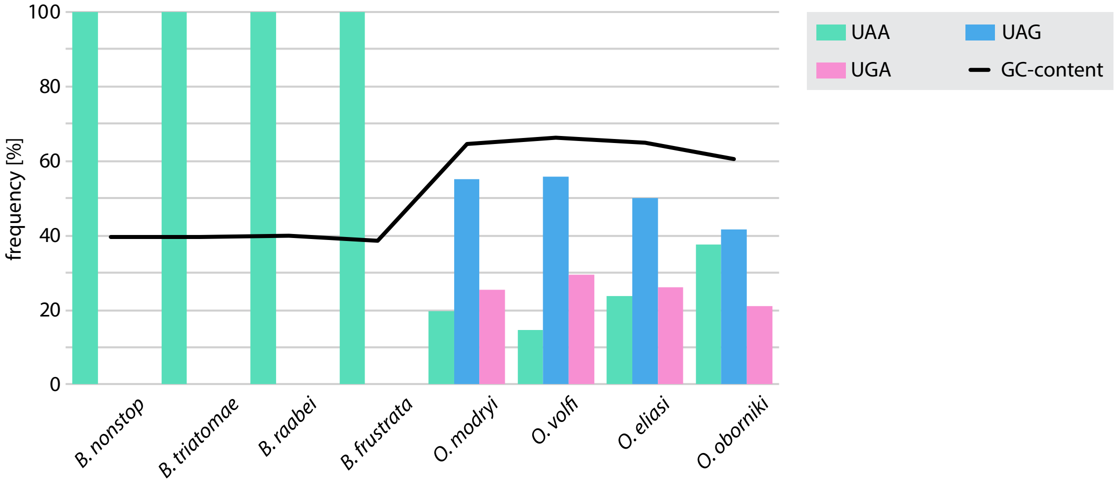

Fig. S13

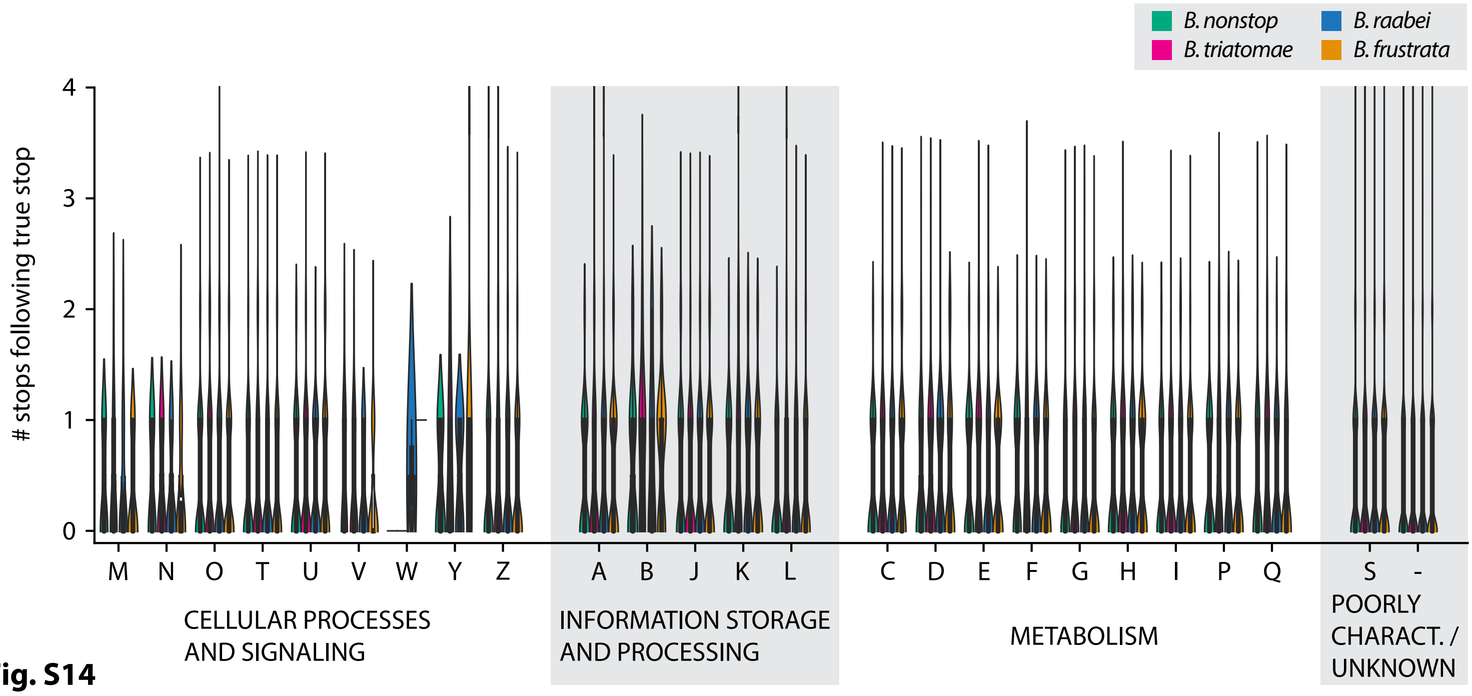

Fig. S14

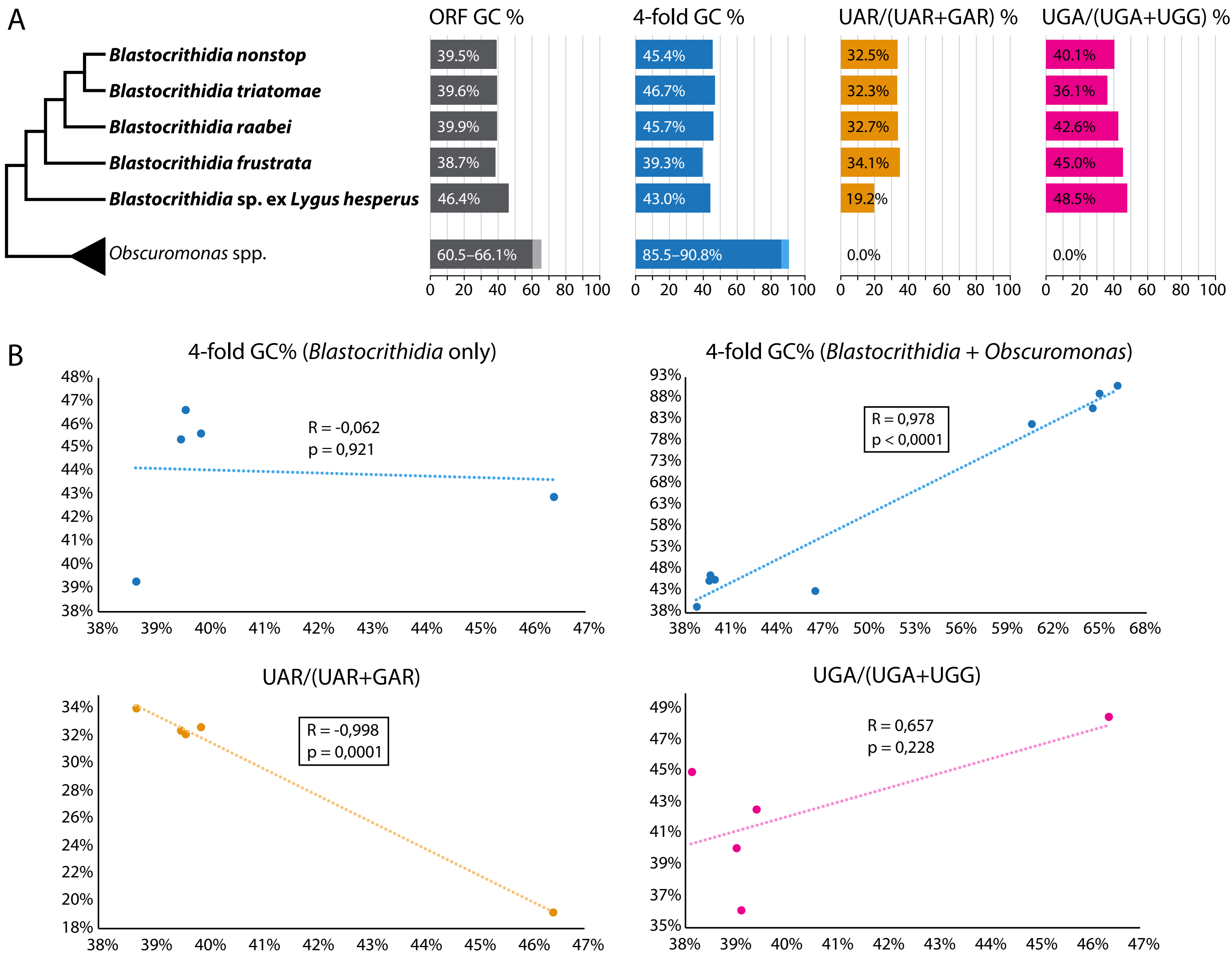

Fig. S15

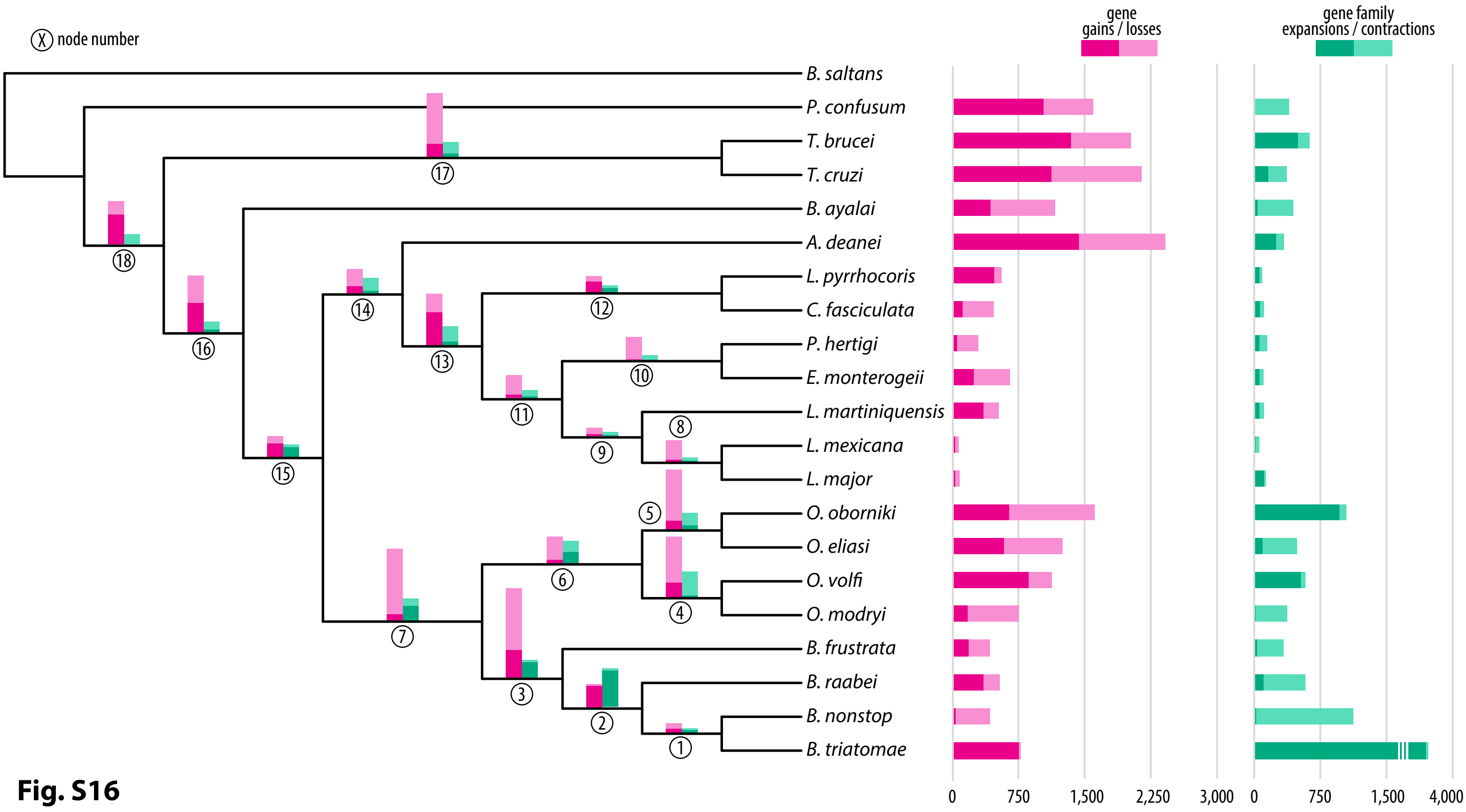

Fig. S16
